# Development and Validation of Novel VHHs Targeting Astrocyte-Enriched Glutamate Transporters GLT-1 and GLAST-1

**DOI:** 10.3390/antib15050089

**Published:** 2026-09-29

**Authors:** Noelia Gesteira-Pérez, Pieterjan Van Maele, Benedetta Frizzi, Ludo Van Den Bosch, Laura Rué, Maarten Dewilde

**Affiliations:** 1 Laboratory for Therapeutic and Diagnostic Antibodies, KU Leuven, 3000 Leuven, Belgium; 2 VIB-KU Leuven Center for Neuroscience, 3000 Leuven, Belgium; 3 Department of Neurosciences, KU Leuven, 3000 Leuven, Belgium; 4 PharmAbs—The KU Leuven Antibody Center, KU Leuven, 3000 Leuven, Belgium

**Keywords:** astrocytes, cell-specific targeting, CNS, drug delivery, GLAST-1/SLC1A3/EAAT1, GLT-1/SLC1A2/EAAT2, glutamate transporters, nanobody/VHH

## Abstract

Background/Objectives. VHHs (Nanobodies^®^) are camelid single-domain antibody fragments with broad applicability in central nervous system (CNS) research, including blood–brain barrier transport, modulation of pathological proteins and molecular detection. However, few VHHs have been developed for cell-type-specific targeting within the brain. Astrocytes play essential roles in CNS homeostasis and contribute to many neurological disorders, making them an appealing target for diagnostic and therapeutic approaches. Here, we report the development and validation of the first VHHs directed against the astrocyte-enriched glutamate transporters GLT-1 and GLAST-1. Methods. VHHs were generated from a camelid immune library using phage display. Target specificity and affinity were evaluated in cells overexpressing the mouse and human transporters, as well as in iPSC-derived astrocytes. Target engagement and astrocyte specificity was further assessed ex vivo on brain sections using immunohistochemistry and in dissociated mouse brain cell suspensions by flow cytometry. VHH in vivo binding was evaluated by stereotactic injections within the brain striatum. Results. One anti-GLT-1 VHH and four anti-GLAST-1 VHHs demonstrated high specificity and apparent nanomolar affinity when binding tocells overexpressing the mouse transporters. The anti-GLT-1 VHH was additionally cross-reactive with the human ortholog, as demonstrated in overexpressing cells and iPSC-derived astrocytes. All VHHs showed target engagement on mouse brain sections and astrocyte specificity in dissociated mouse brain cell suspensions. VHH direct brain delivery resulted in localized transporter-specific signal around the injection site, whereas the non-binding isotype control VHH produced no detectable signal. Conclusions. Here we present the first set of VHHs capable of selectively recognizing astrocyte-enriched glutamate transporters GLT-1 and GLAST-1 in vitro, ex vivo, and in vivo. These VHHs constitute promising tools for astrocyte detection in research applications and the cross-reactive anti-GLT-1 lead might hold potential for future diagnostic or therapeutic strategies, including cell-targeted payload delivery.

## 1. Introduction

Single-domain antigen-binding domains derived from camelid heavy-chain-only antibodies (also known as VHHs or Nanobodies^®^) have emerged over the past decades as versatile affinity binders with several advantages over conventional monoclonal antibodies [1]. Their small size, high specificity, solubility, tissue penetration, remarkable stability, and ease of production and engineering have positioned them as valuable tools across therapeutic, diagnostic, and research applications [2]. Next to promising research tools and potential therapeutics in oncology [3], infectious diseases [4], blood disorders [5] and immunology [6], VHHs are increasingly being explored in the context of central nervous system (CNS) disorders, both as therapeutic agents and as molecular tools for imaging, target detection, or drug delivery [7].

Several VHHs targeting hallmark proteins of neurodegenerative diseases have been reported. Anti-Tau [8,9,10,11,12] and anti-amyloid-β (Aβ) [13,14] VHHs have been developed to detect or reduce Tau tangles or Aβ plaques in Alzheimer’s disease (AD), while anti-α-synuclein VHHs are being investigated for their ability to bind to different forms of aggregate species [15,16] and modulate fibril formation [17,18] in Parkinson’s disease (PD). In parallel, VHHs have been engineered as shuttles to deliver therapeutic cargos across the blood–brain barrier (BBB) via receptor-mediated transcytosis (RMT), using brain endothelial cell enriched targets such as the transferrin receptor (TfR) [19,20,21,22,23], TMEM30a (CD30a) [24,25], insulin-like growth factor 1 receptor (IGF1R) [26,27], or CD98 heavy chain (CD98hc) [28]. Despite these advances, examples of VHHs designed for cell-type-specific targeting within the CNS remain limited.

A small number of studies have described VHHs directed against neuronal targets as potential therapeutic approaches or for imaging purposes [29,30,31,32]. Others have reported VHHs that bind and inhibit the microglial purinergic receptor P2X7 following intracerebral or systemic administration [33,34,35], and additional work has reported the use of a VHH recognizing CD11b (ITGAM) for microglial detection in tissue sections using confocal microscopy [36]. More recently, VHHs with high affinity for myelin oligodendrocyte glycoprotein (MOG) have been developed [37]. For astrocytes, several VHHs against glial fibrillary acidic protein (GFAP) have been reported for ex vivo and in vivo detection [38,39]. However, despite these isolated examples, there are currently no widely adopted VHHs targeting classical pan-cell markers for major CNS cell populations. Developing such tools is essential not only for studying cell-type-specific biology but also for enabling targeted therapeutic delivery, particularly as the contribution of multiple cell types to the onset and progression of neurological disease becomes increasingly recognized [40].

Among the different CNS cell populations, the relevance of astrocytes in brain homeostasis has gained substantial attention in the last decades. Despite having been considered for many years to only act as structural and supporting cells for neuronal function, they are now understood to be key players in health and disease [41,42]. Apart from serving as physical scaffolding to organize and maintain the brain’s architecture, they are in close contact with blood cells to regulate cerebral blood flow, metabolic uptake and BBB maintenance [43]. They participate in the tripartite synapse, modulating synaptic formation, maturation and plasticity [44]. In addition, they guarantee neurotransmitter and ion homeostasis, clear up waste products and contribute to injury responses and repair through glial scar formation and cytokine release [45]. Given that it is now clear that astrocytes and neurons work together to process information, they have been identified to be involved in different physiological processes such as learning, memory, behavior, and even circadian regulation [46]. Moreover, they may drive or exacerbate diseases that had, in the past, been considered to be exclusively neuronal. Numerous studies reported their implication in diverse neurodegenerative diseases such as AD, PD, multiple sclerosis (MS), Huntington’s disease (HD), and neuropsychiatric disorders [47]. Altogether, these findings identify astrocytes as promising therapeutic targets to alleviate different disease symptoms.

Accurate detection and characterization of astrocytes require reliable molecular markers. Several proteins are commonly used to identify astrocytes, including GFAP, S100B, glutamine synthetase (GS), aquaporin-4 (AQP4), glutamate transporter 1 (GLT-1), or glutamate-aspartate transporter 1 (GLAST-1) [48]. GLT-1 and GLAST-1, also referred as excitatory amino acid transporter 2 (EAAT2) and 1 (EAAT1) and encoded by the *SLC1A2* and *SLC1A3* genes, respectively, are the major astrocytic glutamate transporters responsible for clearing extracellular glutamate, thus preventing excitotoxicity [49]. Although there are other glutamate transporters expressed in neurons, GLT-1 and GLAST-1 are predominantly expressed in astrocytes [50]. GLT-1 in fact accounts for over 90% of total synaptic glutamate clearance and it has been regarded as a pan-astrocytic marker [51]. Because of their crucial function in neurotransmission, the dysregulation of GLT-1 and GLAST-1 has been linked to excitotoxicity and neuronal death, contributing to the pathology of several neurological disorders, including AD, PD, HD, amyotrophic lateral sclerosis (ALS) and epilepsy [52,53,54].

Given the central role of astrocytic glutamate transporters in CNS physiology and disease, tools enabling their precise detection are of significant value. To investigate the expression, localization and regulation of these transporters, early studies report the development of both anti-GLT-1 and GLAST-1 antibodies. The purification, and subsequent cloning and expression of, for the first time, rat GLT-1 [55] enabled the generation of different monoclonal [56,57] and polyclonal [58,59] antibodies against this transporter, which have been further characterized using different techniques, sample types and species, ranging from human tissues to knock-out mouse models [60,61,62,63,64,65]. In parallel, and in a similar fashion, antibodies against this transporter also became available [58,59,66,67] after the cloning of rat GLAST-1 [68], and were further validated by different authors [62,64,69,70]. In addition to this, there are currently several commercially available antibodies against both targets, which are extensively used for various research applications. All of these tools have unarguably allowed for the elucidation of different aspects related to the transporters’ expression, to (sub)cellular localization and tissue biodistribution, as well as to the study of their function and activity in physiological conditions and different disease contexts [65,66,71,72,73]. Collectively, these studies highlight the continued relevance of targeting these transporters and support the development of additional antibody modalities. However, most of the published antibodies were raised against intracellular epitopes [70], which represents a limitation for certain experimental techniques that require binding to the native transporter on the surface of intact, non-permeabilized cells, or even for therapeutic applications. In this context, VHHs represent promising supplementary tools for the detection of complex protein targets, offering several advantages over conventional antibodies, including smaller size, modularity, enhanced stability, and improved tissue penetration [2].

In this study, we describe and characterize what, to the best of our knowledge, are the first VHHs targeting GLT-1 and GLAST-1. We demonstrate their utility as molecular tools for the detection of astrocytes in mouse, and potentially human, brain samples using multiple experimental approaches.

## 2. Materials and Methods

### 2.1. Target Identification by Database Mining

Public single-cell (sc) sequencing databases, such as The Human Protein Atlas (HPA), Brain RNA-Seq or mousebrain.org, were mined in order to select targets enriched in astrocytes. Different criteria were applied to establish a ranking of different proteins considering targetability (high, cell surface expression in mouse and human brain, especially in astrocytes) and feasibility from a VHH development point of view (ideally aiming for candidates with high sequence conservation between mouse and human, for clinical translation, and lower with camelids, for effective immunization).

### 2.2. Immunization and VHH Library Preparation

One alpaca and one llama were subjected to a campaign of four bi-weekly DNA immunizations (Lamasté, Mol, Belgium), using a pool of recombinant pVAX1 plasmids encoding various proteins, including the longest mRNA transcript variant of mouse *Slc1a2* (UniProt #P43006) and *Slc1a3* (UniProt #P56564), synthetized by Twist Bioscience (San Francisco, CA, USA). The DNA pool was injected intradermally at multiple sites at the front and back limbs near the draining lymph nodes, followed by electroporation. A total amount of 2 mg of the plasmid DNA pool was used per injection per animal. On days 4 and 8 after the last immunization, 100 mL of anticoagulated blood was collected from each animal for the preparation of peripheral blood mononuclear cells (PBMCs). The VHH-encoding genes were recovered and the phagemid library was cloned as previously described [74]. In short, total RNA from PBMCs was used as template for first-strand cDNA synthesis with oligodT primers. This cDNA was used to amplify the VHH-encoding open reading frames by polymerase chain reaction (PCR), digested with *PstI* and *NotI* (Thermo Fisher Scientific, Waltham, MA, USA), and cloned into a phagemid vector. The library was transformed into electro-competent *Escherichia coli* (*E. coli*) TG1 cells (Biosearch Technologies, Middlesex, UK), and phage libraries were generated by superinfection with M13KO7 helper phage (Thermo Fisher Scientific).

### 2.3. Generation of Target Overexpressing Cell Lines

Chinese hamster ovary (CHO) cell lines, stably overexpressing mouse *Slc1a2* and *Slc1a3* were generated with the Flp-In™ CHO™ system (Invitrogen™, Thermo Fisher Scientific). DNA encoding for these two genes was subcloned from the pVAX1 into the pcDNA™5/FRT mammalian expression vector containing the green fluorescent protein (GFP) gene under the control of an internal ribosome entry site (IRES) downstream of the target gene. The target expressing vectors and the Flp-In™ recombinase vector pOG44, were co-transfected in the Flp-In™ CHO™ cell line using X-tremeGENE 9 DNA transfection reagent (Sigma-Aldrich, St. Louis, MO, USA). Cells were cultured in Ham’s F-12 Nutrient Mix medium supplemented with GlutaMAX (Gibco™, Thermo Fisher Scientific), 10% fetal bovine serum (FBS) (Gibco™) and Zeocin™ selection antibiotic (100 µg/mL, Invivogen™, San Diego, CA, USA) before transfection, or Hygromycin B gold (700 µg/mL, Invivogen™) after transfection, to select for stably transfected cells.

Human embryonic kidney (HEK) 293 suspension cells were transiently transfected with the pcDNA™5/FRT mouse-*Slc1a3*-containing vector using 293fectin™ Transfection Reagent (Gibco™), according to the manufacturer’s protocol, to generate overexpressing cells (HEK*^Slc1a3^*). Briefly, cells were grown and expanded in baffled flasks containing FreeStyle™ 293 Expression Medium (37 °C, 95% relative humidity, 5% CO_2_, 125 rpm) until transfection. Right after transfection, they were cultured at 8% CO_2_ for 48 h and frozen with FBS-supplemented medium containing 10% dimethyl sulfoxide (DMSO) (Sigma-Aldrich) in liquid nitrogen for further use.

HEK293 Jump In^TM^ T-REx^TM^ (Thermo Fisher Scientific) and HCT116 cell lines with the wild type (WT), knock out (KO) and/or overexpresed (OE) human ortholog were kindly donated by the RESOLUTE consortium [75]. HEK*^SLC1A2^* WT-OE, HEK*^SLC1A3^* WT-OE, HCT116*^SLC1A3^* KO, and HCT116*^SLC1A3^* KO-OE cell lines were generated following different protocols described by RESOLUTE. Once received, HEK cells were cultured in Dulbecco’s-modified Eagle medium (DMEM) supplemented with GlutaMAX, 10% FBS and 2 mg/mL geneticin (Gibco™) and 5 μg/mL blasticidin (Merck, Rahway, NJ, USA) as selection antibiotics. HCT116 cells were cultured in RPMI 1640 medium with L-glutamine (Gibco™) supplemented with 10% FBS. For HCT116*^SLC1A3^* KO-OE cell line, 10 µg/mL blasticidin (Merck) was used as selection antibiotic. To induce gene expression in all OE cell lines, 1 μg/mL doxycycline (Dox) (Sigma-Aldrich) was added for 24 h, after which they were harvested. Dox derepresses the promoter upstream of the gene of interest (GOI), thus allowing its expression.

All adherent cell lines used were cultured and maintained at 37 °C in a humidified incubator (95% relative humidity) with 5% CO_2_. They were passaged with 0.05% trypsin-ethylenediaminetetraacetic acid (EDTA) (Gibco™) harvested with 0.5 mM EDTA (Invitrogen™) and frozen with the corresponding FBS-supplemented cell medium containing 10% DMSO in liquid nitrogen. Validation of target expression in all cell lines was performed by flow cytometry prior to further use (Figure S2).

### 2.4. VHH Selection, Expression and Purification

In order to identify anti-GLT-1 and anti-GLAST-1 VHHs, VHH-displaying M13 phage libraries were prepared according to standard protocols [74] and selected on CHO*^Slc1a2^* or CHO*^Slc1a3^* cells, respectively. Briefly, 4 × 10^11^ cfu of phages were blocked with PBS/10% FBS and incubated (1 h, 4 °C, rotating) with 5  ×  10^6^ cells in each round. GLT-1/SLC1A2 pannings consisted of three selection rounds on CHO*^Slc1a2^* cells, whereas, for GLAST-1/SLC1A3, a pre-depletion was performed before the first round using HCT116*^SLC1A3^* KO cells, after which three rounds of pannings were performed on CHO*^Slc1a3^* cells. Non-binding phages were discarded with 5 consecutive washing steps with PBS/10% FBS, whereas bound phages were eluted by means of trypsinization. The enriched output phage library of the third selection round was subcloned into an expression vector (a modified pHEN6 vector with an OmpA signal peptide and a C-terminal 3×FLAG/6×HIS tag) using *MfeI* and *Eco91L* restriction enzymes (Thermo Fisher Scientific) and transformed in TG1 *E.coli* cells for VHH expression from single colonies. Small-scale expression from single clones was performed and periplasmic extracts were prepared in two 96-well plates as previously described [74] and screened for direct binding to CHO*^Slc1a2^* or CHO*^Slc1a3^* cells. Identified hits were sequenced (LGC Genomics, Berlin, Germany) and clustered according to sequence homology with PipeBio cloud-based bioinformatics platform in 2023 (Benchling, San Francisco, CA, USA). After this, selected VHHs (1 anti-GLT-1 and 9 anti-GLAST-1) were expressed following the protocol by Pardon et al. [74]. Purification was done using AmMag™ Ni-charged magnetic beads and the AmMag™ SA Plus System (Genscript, Piscataway, NJ, USA).

### 2.5. VHH In Vitro Screening and Affinity Determination

Periplasmic extracts diluted 1:10 or a dilution range of different VHH concentrations prepared in staining buffer (PBS/2% FBS) were screened for direct binding on CHO*^Slc1a2^*, CHO*^Slc1a3^*, HEK*^Slc1a3^*, Dox-induced HEK*^SLC1A2^* or HCT116*^SLC1A3^* KO-OE cells using flow cytometry. Briefly, constructs were incubated in a 96-well plate with 10^5^ cells per well (30 min, on ice) after which they were washed twice with staining buffer and stained with a mouse anti-FLAG-iF647 (Table S2) and eBioscience Fixable Viability Dye eFluor780 (1:2000, Cat. No. 65-0865-14, Invitrogen™) in staining buffer (30 min, on ice, protected from light), for VHH detection and live/dead staining, respectively. Next, cells were washed once with staining buffer and finally resuspended in the same buffer. Flp-In™ CHO™ cells, used as unstained control, and single stain controls were used to determine the cutoff value between background fluorescence and positive populations. UltraComp eBeads™ compensation beads (Thermo Fisher Scientific) were used to generate a single-stain control of anti-FLAG-iF647 antibody. Data were acquired using an Attune NxT flow cytometer (Thermo Fisher Scientific) and analyzed by FCS Express 7 (De Novo Software, Pasadena, CA, USA).

### 2.6. VHH:Fc Fusions Expression and Purification

Plasmid DNA (pDNA) containing the VHH:Fc fusion encoding genes were synthesized by Twist Bioscience. Once received, plasmids were amplified in *E. coli* TOP10F’ and purified with NucleoBond Xtra Maxi EF kit (Machery-Nagel, Düren, Germany). pDNA concentration and purity were determined via UV spectrophotometry and agarose gel electrophoresis, respectively, and VHH:Fc DNA sequences were confirmed through Sanger sequencing (LGC genomics).

VHH:Fc fusions were expressed using ExpiCHO-S™ cells (Thermo Fisher Scientific) and the Mirus CHOgro^®^ expression system (Mirus Bio, Madison, WI, USA) according to the manufacturer’s protocol. Briefly, ExpiCHO-S™ cells were grown in CHOgro^®^ expression medium until reaching the desired numbers. For each transfection, pDNA was mixed and incubated (5 min, RT) with TransIT-PRO^®^ transfection reagent in CHOgro^®^ complex formation solution before being added to the cell culture. Immediately after transfection, CHOgro^®^ titer enhancer was added directly to the cultures and they were incubated (32 °C, 8% CO_2_, 125 rpm) for 14 days for protein expression.

VHH:Fc purification from cell medium was done using AmMag™ Protein A magnetic beads and the AmMag™ SA Plus system according to the manufacturer’s protocol. Following elution with 0.1 M glycine buffer (pH = 2–3) and immediate neutralization to pH = 7 with 1 M Tris buffer pH = 8.5, buffer exchange to PBS was carried out by dialysis using membranes with a molecular weight cut-off (MWCO) of 12 kDa (Medicell Membranes Ltd., London, UK). Proteins were then concentrated using Vivaspin^®^ Turbo centrifugal concentrator columns 10 kDa MWCO (Cat. No. VS15T01, Sartorius, Göttingen, Germany). VHH:Fc purity and aggregation were quality controlled by sodium dodecyl sulfate-polyacrylamide gel electrophoresis (SDS-PAGE) and size exclusion chromatography (SEC), respectively, using a Superdex 200 Increase 10/300 GL column and the Akta system (Cytiva, Marblorough, MA, USA). Target binding retention was demonstrated in mouse and human GLT-1 overexpressing cells as described in Section 2.5.

### 2.7. Animals

All animal experiments were conducted in compliance with the commonly accepted ‘3Rs’ and approved by the local Ethical Committee of Laboratory Animals of the KU Leuven (governmental license LA1210261, ECD project number DFFW-P107/2024, approval date: 23 July 2024), following governmental and EU guidelines. Mice were housed under standard conditions according to the guidelines of KU Leuven, with a 12 h light/dark cycle and with access to food and water ad libitum. The ex- and in vivo experiments were performed using 7–9 weeks old male and female WT C57BL/6 mice.

### 2.8. VHH Binding Validation in Mouse Brain Homogenates by Flow Cytometry

Mice (*n* = 3) were euthanized with a Dolethal^®^ (pentobarbital) (Vetoquinol, Lure, France) overdose (150–200 mg/kg) injected peritoneally. Brains were harvested after transcardial perfusion with PBS/heparin (5 U/mL, Leo Pharma, Ballerup, Denmark) and the two hemispheres were transferred separately to two different tubes containing staining buffer (PBS/2% FBS/2 mM EDTA). Each hemibrain was enzymatically and mechanically dissociated using a neural tissue dissociation kit (T) or (P) (Cat. No. 130-093-231 or 130-092-628, Miltenyi Biotech, Bergisch Gladbach, Germany) following the manufacturer’s recommendations. Briefly, brain hemispheres were transferred to the corresponding enzyme mix, cut into small pieces using Noye spring scissors (Cat. No. 15012-12, Fine Science Tools, Foster City, CA, USA) and incubated for 15 min at 37 °C while shaking. After, tissues were dissociated using a P1000 micropipette with 3 sets of tips cut at different sizes followed by incubation with the next enzyme mix (10 min, 37 °C, shaking). After this, the cell suspension was mixed again using a P1000 and filtered using a 70 μm cell strainer. Cells were then washed once with staining buffer, centrifuged (300× *g*, 4 °C, 15 min), and the pellet resuspended in a PBS/24% Percoll™ solution (Cat. No. 17-5445-02, GE Healthcare, Chicago, IL, USA) for myelin removal. After centrifugation (300× *g*, 4 °C, 15 min), supernatant was carefully discarded and the obtained cell pellets were immediately used for flow cytometry staining.

Fresh mouse brain cell suspensions were, prior to staining, incubated (10 min, on ice) with a fragment crystallizable region receptor (FcR) blocking reagent (1:10, Cat. No. 130-092-575, Miltenyi Biotech). After washing with staining buffer and centrifuging (300× *g*, 4 °C, 10 min), cells were plated into a V-bottom 96-deep-well plate and incubated (30 min, on ice, protected from light) with VHHs (100 nM) binding to different targets (GLT-1, GLAST-1, MOG and β-lactamase) and anti-cell marker antibodies, in order to simultaneously detect the presence of astrocytes, microglia, and oligodendrocytes (Table S1) together with an eBioscience Fixable Viability Dye eFluor780 viability dye (1:2000). Following incubation, cells were washed with staining buffer, centrifuged (300× *g*, 4 °C, 5 min) and pellets were stained (30 min, on ice, protected from light) with a mouse anti-FLAG-iF488 antibody (Table S2) to detect VHH binding. Cells were then washed and finally resuspended in staining buffer. Attune NxT flow cytometer was used for data acquisition and analysis was performed using FCS Express 7.

### 2.9. Stereotaxic Intracerebral Injections

Anti-GLT-1, GLAST-1 or β-lactamase VHHs were directly injected into the striatum of mice brains (*n* = 1/group) by using a stereotaxic frame. Briefly, mice were injected subcutaneously (SC) with Metacam^®^ (5 mg/kg; Boehringer Ingelheim, Ingelheim am Rhein, Germany) at least 30 min prior to surgery as general analgesics. Mice were anesthetized with isoflurane (1000 mg/g, Iso-Vet; Piramal Critical Care, Voorschoten, The Netherlands) (3% for induction, 1–2% for maintenance) in 2.5 L/min O_2_ supply. After placing them in the stereotaxic frame (WPI, Sarasota, FL, USA), a 1:1 mix of Xylocaine 1%^®^/Marcaine 0.5%^®^ (Aspen, Denver, CO, USA) was injected SC under the scalp as local analgesia 5 min before a 1-cm-long incision above the midline was made. A cranial burr hole (1.0 mm) was drilled at the coordinates AP + 0.86 mm, ML ± 1.5 mm, and DV − 3.0 mm (from bregma). VHHs (140 µM in 2 ul of PBS) were loaded into a 10 μL Hamilton syringe (Cat. No. 1701RN, Hamilton, Reno, NV, USA) connected to a 26-gauge needle (Cat. No. 7804-03, Hamilton) controlled by a motorized stereotaxic injector (WPI). The needle was slowly introduced to the desired depth and after 1 min the subjects were injected at 0.25 μL/min. This step was followed by a 5 min break to allow diffusion and slow removal of the needle. Body temperature was maintained throughout the procedure at 37 °C using a homeothermic monitoring system (Harvard Apparatus, Holliston, MA, USA). At the end of the surgery, the cranial hole was closed using bone wax (Cat. No. 901, Surgical Specialities, Westwood, MA, USA), the skin incision was stitched, and NaCl 0.9% (0.2 mL/10 g, B. Braun, Melsungen, Germany) was injected SC at the back of the mice to prevent dehydration.

### 2.10. Immunohistochemistry and Imaging

Non-injected or VHH-injected brains were harvested 24 h post-treatment after transcardial perfusion with heparinized PBS and post-fixed by immersion in 4% PFA (24 h, 4 °C) (Sigma). After this, they were stored at 4 °C in PBS/0.02% sodium azide (Sigma) until further use. On the day of sectioning, brains were embedded in PBS/4% low melting point agarose (Thermo Fisher Scientific) and coronal or sagittal sections of 30 µm were obtained using a Leica VT1000S vibratome (Leica, Wetzlar, Germany) and stored in PBS/0.02% sodium azide until immunohistochemistry (IHC) analysis. For IHC, brain slices were placed on Superfrost plus slides (Thermo Fisher Scientific), washed once with PBS and immediately blocked and permeabilized in blocking buffer (PBS/5% goat serum [Sigma]/0.1% Triton-X 100 [Sigma]) (1 h, room temperature [RT]), followed by staining with different VHHs (GLT-1, GLAST-1 or β-lactamase) or primary antibodies (Table S2) diluted in blocking buffer (ON, 4 °C). The next day, tissues were washed 3 times in PBS/0.1% Triton-X 100 and incubated with secondary antibodies (Table S2) and Hoechst (1:5000; Sigma) (2 h, RT, protected from light). After 3 washes, prolong gold anti-fade mounting medium (Thermo Fisher Scientific) was added to the sections and a coverslip was placed on top. Images were collected with a ZEISS Axioscan 7 at 20× objective (ZEISS, Oberkochen, Germany) and processed using ZEN software version 3.13 (ZEISS).

## 3. Results

### 3.1. GLT-1 and GLAST-1 Selection as VHH Targets for Astrocyte Detection

Candidate targets enriched in astrocytes were identified through a rational database-mining strategy designed to maximize both targetability and suitability for VHH development. Thousands of genes from publicly available scRNA-sequencing datasets were screened using certain predefined criteria. First, we selected genes expressed in both mouse and human to support potential translational use of the resulting VHHs while guaranteeing pre-clinical development in mouse models. We then restricted the list to (trans)membrane proteins, given that VHHs are generally not internalized without additional engineering and therefore require accessible extracellular epitopes [76]. To ensure astrocyte specificity, we prioritized genes with >50% expression enrichment in astrocytes relative to other brain cell populations (Figure S1). In parallel, we favored targets with high expression in the brain and limited expression in peripheral organs to minimize potential off-target effects during systemic in vivo delivery. From a VHH-development perspective, we further selected proteins with sufficiently large extracellular domains and high sequence homology between mouse and human to increase the likelihood of generating cross-reactive VHHs (Figure S2A–C). Low sequence similarity between mouse and alpaca orthologs was also considered for an efficient immune response during the immunization campaign; however, most of the targets showed high homology with the camelid ortholog. Based on these combined criteria, two mouse genes, *Slc1a2* and *Slc1a3*, were selected, encoding for the multispan glutamate transporters GLT-1/EAAT2 and GLAST-1/EAAT1, respectively. These ranked among the top candidates due to their high expression in the brain, strong astrocytic specificity, and >90% coding-sequence homology between mouse and human (Table 1; Figure S2B,C).

### 3.2. Biopannings and Identification of Anti-GLT-1 and Anti-GLAST-1 VHHs

To obtain anti-GLT-1 and GLAST-1 VHHs, two camelids were immunized using DNA encoding the mouse genes (*Slc1a2* and *Slc1a3*, respectively). The construction of the VHH phage library was based on the protocol published by Pardon et al. [74], illustrated in Figure 1A. Two different libraries were generated, one per animal, with estimated sizes of 1.32 × 10^8^ cfu (llama) and 2.76 × 10^7^ cfu (alpaca) and insert rates of ~74% and ~80%, respectively. In order to identify VHHs specifically binding to GLT-1 and GLAST-1, the two VHH-displaying phage libraries were separately subjected to three in vitro selection rounds on the CHO*^Slc1a2^* or CHO*^Slc1a3^* cell lines, respectively. The output libraries were used to transform *E. coli* TG1 cells and single clones were randomly picked and expressed as periplasmic extracts in a 96-well format. Screening results on CHO*^mGOI^* cells using flow cytometry revealed one potential binder for mouse GLT-1 and 18 for GLAST-1. Hits were sequenced, which resulted in one VHH sequence for GLT-1 and eight sequentially unrelated VHH clusters for GLAST-1. One VHH for GLT-1 (P00400001) and eight VHHs for GLAST-1 (P00400003-10) were expressed (containing C-terminal 3×FLAG/6×HIS tags) and purified for further characterization (Figure 1B).

### 3.3. In Vitro Anti-GLT-1 and Anti-GLAST-1 VHHs Binding to Their Mouse and Human Targets

The in vitro binding properties of the nine selected VHHs were evaluated by flow cytometry using CHO*^Slc1a2^* and CHO*^Slc1a3^* or HEK*^Slc1a3^* cell lines. Serial dilutions of each pure VHH were incubated with the cells, and binding was detected using an anti-FLAG-iF647 antibody. For GLT-1, the VHH tested demonstrated specific binding to its mouse’s intended target, while for GLAST-1 only four out of eight VHHs bound (Figure S4). The isotype control VHH targeting β-lactamase [77] (a bacterial enzyme not expressed in mammalian cells) did not bind to either cell line, as expected (Figure 2A,B). All VHH binders displayed apparent affinities to the mouse target overexpressed on cells within the nanomolar range, with some VHH-dependent variation (Table S3).

To determine whether the anti-GLT-1 and anti-GLAST-1 VHHs were able to recognize the corresponding human orthologs, binding assays were performed using Dox-induced HEK*^SLC1A2^* WT-OE and HCT116*^SLC1A3^* KO-OE cell lines. The anti-GLT-1 VHH was the only candidate showing cross-reactivity with human GLT-1 (Figure 2C and Figure S3C), whereas none of the GLAST-1-reactive VHHs recognized the human ortholog (Figure 2D and Figure S3D). Given the similarities in structure, i.e., highly conserved overall fold and transporter architecture, and ~50% sequence identity among members within the EAAT family [78,79], including GLT-1 (*SLC1A2*) and GLAST-1 (*SLC1A3*) (Figure S2A,D), potential VHH cross-reactivity was assessed for both mouse and human; however, none of the VHH leads exhibited binding to the other transporter, further supporting their target specificity (Figures S4 and S5).

To further validate binding to human GLT-1 in a more physiological model, human induced pluripotent stem cells (hiPSC) were derived from somatic cells and differentiated into mature astrocytes using previously described protocols [80,81]. Because GLT-1 expression can vary according to differentiation protocol and maturation stage, transporter levels in each biological replicate were confirmed by Western blot (WB) prior to binding studies, revealing similar protein levels for differentiations #1 and #2 while slightly lower for the #3 (Figure S7A). Astrocytes were then incubated with a commercial antibody against GLT-1, which confirmed GLT-1 cell membrane expression on hiPSC astrocytes by flow cytometry (Figure S7B). Next, the same experiment was performed by incubating the cells with two concentrations (30 and 300 nM) of the anti-GLT-1 or the isotype control VHH in a monovalent or bivalent format, generated by genetic fusion to the Fc region of a human IgG (VHH:Fc). VHH:Fc ability to bind to both mouse and human GLT-1 was re-confirmed in overexpressing cells prior to this experiment (Figure S6). While no anti-GLT-1 VHH binding was detected in a monovalent format in hiPSC astrocytes, giving comparable signals to the isotype control VHH (Figure S7C), incubation with the anti-GLT-1 VHH:Fc showed dose-dependent binding (Figure S7D), obtaining lower signals for differentiation #3, which correlates with what was previously observed by WB.

Collectively, these data demonstrate the specific binding of one VHH to both mouse as well as human GLT-1, and four VHHs to mouse GLAST-1 without cross-reactivity to the human ortholog.

### 3.4. Anti-GLT-1 and Anti-GLAST-1 VHHs Showed Specificity Towards Astrocytes in Mouse Brain Homogenates

Having identified anti-GLT-1 and anti-GLAST-1 VHHs and validated their specificity in target-overexpressing in vitro cell models, we next assessed whether their binding capacity was retained in mouse primary cells expressing endogenous transporter levels.

For this purpose, ex vivo binding studies were performed using brains from WT C57BL/6 mice. Tissues were enzymatically and mechanically dissociated using both papain- and trypsin-based protocols, enabling evaluation of VHH binding across some of the major brain cell populations. Astrocytes, oligodendrocytes, and microglia were identified by flow cytometry using antibodies against ACSA-2, O1, and CD11b, respectively (Figure 3A). Both dissociation strategies yielded sufficient numbers of viable cells, and comparable proportions of astrocytes (ACSA2^+^O1^−^), oligodendrocytes (O1^+^), and microglia (CD11b^+^) were recovered across animals, with some variation between dissociation methods (Figure S8A).

Incubation of trypsin-dissociated brain homogenates with 100 nM anti-GLT-1 VHH (P00400001) resulted in predominant binding to astrocytes, with >60% of astrocytic cells staining positive, compared with <10% microglia and <4% oligodendrocytes, as measured by both percentage of VHH-positive cells (% VHH^+^) and median fluorescence intensity (MFI) (Figure 3B). Similarly, anti-GLAST-1 VHHs (P00400003/8/9/10) also showed clear enrichment towards the astrocytic population, with >70% of astrocytes stained across all VHHs, although with construct-dependent variability in MFI (Figure 3C). In contrast, when brain tissue was dissociated with papain, only the anti-GLT-1 VHH displayed detectable binding (Figure S8C,D).

Control samples, including the β-lactamase-specific VHH (LTDA00021) or VHH-detection antibody only, showed no detectable binding, further confirming assay specificity (Figure 3B,C). As a positive control, an in-house-developed VHH targeting MOG (BBB00498) [37] was included. As expected, this construct bound primarily to oligodendrocytes (>40%), validating the sensitivity and integrity of the dissociated cell preparations (Figure S8B).

Altogether, these results demonstrate that anti-GLT-1 and anti-GLAST-1 VHHs retain the ability to recognize their respective endogenous targets in primary mouse brain cells and support their use as astrocyte-selective binders in ex vivo applications.

### 3.5. Anti-GLT-1 and Anti-GLAST-1 VHH Bound to Their Target Ex Vivo in Mouse Brains Tissues by IHC

To evaluate whether anti-GLT-1 and anti-GLAST-1 VHHs could be used as immunohistochemical tools to detect their respective targets in mouse tissue, they were applied at 1 µM to sagittal brain sections from WT C57BL/6 and detected using a mouse anti-HIS-iF647 antibody (Figure 4A). Whole-brain scans revealed broad GLT-1 and GLAST-1 distribution, with regional differences in signal intensity consistent with known expression patterns, including high expression in astrocytic processes and prominent GLAST-1 enrichment in Bergmann glia within the cerebellar molecular layer. In contrast, incubation with the isotype control VHH produced only minimal background staining, comparable to the no-VHH control, confirming the absence of non-specific binding (Figure 4B,C).

To further assess target specificity, co-staining with commercial antibodies against GLT-1 or GLAST-1 were performed to check for colocalization between the two different affinity binders. The staining patterns obtained with VHHs closely matched those produced by the corresponding antibodies (Figure 4C). Both glutamate transporters displayed similar localization profiles, with strong enrichment in the neuropil across multiple brain regions. Qualitative assessment of VHH and antibody signals demonstrated clear colocalization, supporting the conclusion that the VHH-derived signal results from specific recognition of the intended transporter (Figure 4D).

### 3.6. Anti-GLT-1 and Anti-GLAST-1 VHH Bound to Their Target In Vivo

After demonstrating target specificity of the different VHHs in vitro and ex vivo, we next assessed whether their binding capacity was preserved in an in vivo context. Given that both transporters showed widespread expression across the brain in ex vivo assays, we selected the dorsomedial striatum for local VHH delivery, which, being a large region, allows reproducible stereotaxic injections, minimizes the risk of ventricular leakage, and facilitates visualization of VHH spread.

Anti-GLT-1, anti-GLAST-1 (P00400009), or anti-β-lactamase VHHs were unilaterally injected at 140 µM (~5 µg), and brains were collected 24 h later for immunofluorescence analysis (Figure 5A). Both anti-GLT-1 and anti-GLAST-1 VHHs were detectable in the brain after 24 h, with signal localized to regions surrounding the injection site. In contrast, the isotype control VHH produced minimal signal at the same time point (Figure 5B). The neuropil-like staining patterns observed for both anti-transporter VHHs were consistent with those obtained in the ex vivo experiments (Figure 4D), further supporting in vivo target engagement (Figure 5C).

## 4. Discussion

VHHs have become increasingly valuable across research, diagnostic, and therapeutic applications due to their unique biochemical and biophysical properties [2]. Their small size enhances tissue penetration and reduces steric hindrance in dense cellular environments, enabling superior performance and improved spatial resolution in applications like imaging or the delivery of therapeutic cargos to solid tissues [82,83]. Their exceptional stability under harsh conditions further supports their use in diverse detection platforms, including ELISA, lateral flow assays, and biosensors [84]. In addition, their modularity and ease of engineering allow the generation of multivalent or multispecific constructs as well as the conjugation of VHHs to drugs, toxins, or nanoparticles, broadening their utility in targeted delivery strategies [3]. All of these properties position them as alternative or complementary tools to already existing antibodies for research purposes or therapeutic applications.

Here, we report the first VHHs, to our knowledge, directed against the astrocytic glutamate transporters GLT-1 and GLAST-1. Although monoclonal and polyclonal antibodies against these transporters are commercially available or have been previously developed by other research groups [58,59], at time of writing no VHH-based affinity binders have previously been described. In this study we therefore sought to develop and characterize them as tools for detecting astrocytes using different in vitro, ex vivo, and in vivo assays.

VHH development for this study relied on a cDNA-based camelid immunization strategy. This approach was selected because the two target proteins are multispan transmembrane glutamate transporters with complex tertiary structures, which makes in-house or commercial production and purification of high-quality recombinant protein material for conventional immunization challenging [85]. Only one anti-GLT-1 and four unique anti-GLAST-1 VHH binders could be identified, underscoring the inherent difficulty of generating affinity binders against structurally complex membrane proteins. Although genetic immunization is expected to allow antigen presentation in a native conformation in the host cell’s membrane, low and transient target expression levels may limit the efficiency of this strategy [86]. In addition, although during our target identification workflow we ideally aimed for the selection of candidates with low sequence homology with camelids, this proved challenging. The high degree of sequence conservation within the extracellular domains of the alpaca and mouse proteins (>90%) might have likely contributed to a reduced immune response. To potentially improve the number of antigen-specific clones, DNA immunization could be combined with other antigen formats, such as cells overexpressing the target protein [86]. Furthermore, the in silico de novo design of VHHs represents an emerging alternative strategy for generating binders against complex membrane proteins [87].

In vitro characterization of the VHH hits using cells overexpressing the mouse transporters identified one GLT-1-specific and four GLAST-1-specific VHHs, all exhibiting apparent affinities in the nanomolar range. It is worth mentioning that the affinities reported were derived from binding experiments on cells expressing the transporters in their native membrane environment, and are therefore reported as apparent affinities. Determination of kinetic binding constants for GLT-1 and GLAST-1 would require reconstitution strategies that preserve transporter conformation and functionality, which was beyond the scope of the present proof-of-concept study. Another important aspect that remains to be investigated is whether VHH binding is influenced by the conformational state of GLT-1 and GLAST-1, as both transporters undergo substantial conformational rearrangements during substrate transport [88]. As the present study was primarily focused on VHH discovery and validation, we did not assess the impact of transporter activity, substrate occupancy, or pharmacological modulation on VHH binding. Future studies evaluating VHH recognition under different functional states may provide additional insight into binding robustness under physiologically relevant conditions, and could further contribute to identification of the underlying epitopes recognized by these VHHs.

Cross-reactivity analysis revealed that only the GLT-1-targeting VHH recognized the human ortholog. Although we selected candidates presenting high sequence homology between mouse and human during the database mining process for target identification, interspecies differences in GLAST-1 extracellular domains, combined with the use of mouse antigens for immunization, might have contributed to the absence of cross-species recognition. This suggests that all four VHHs tested might recognize an epitope containing species-specific sequence features. In fact, comparison of the mouse and human GLAST-1 sequences revealed a few aminoacidic differences within different extracellular regions that are accessible to VHH binding. Although the present data do not allow definitive epitope assignment, these sequence differences may represent a potential explanation to the observed species specificity. Future studies combining targeted mutagenesis, domain-swapping approaches and/or structural characterization will be required to identify the precise VHH binding epitope for all of the identified leads. Moreover, future immunization or boosting strategies incorporating human antigens, as well as biopanning on human target-expressing cells, may increase the likelihood of isolating cross-reactive VHHs. Nonetheless, a notable strength of our VHHs lies in the strategy used for their identification and validation. By combining cDNA immunization, cell-based panning, and live-cell surface binding assays, the full-length transporters were expressed in a native membrane environment. Therefore, we are confident that the selected VHHs recognize extracellularly accessible regions of the transporters, although format epitope mapping/binning studies have not yet been performed. Extracellular binding may represent a significant advantage over many previously published anti-GLT-1 and anti-GLAST antibodies, which are predominantly directed against intracellular N- or C-terminal domains [70]. As a result, our VHHs may offer superior suitability for applications requiring extracellular target engagement, including live-cell studies, imaging or functional assays. More importantly, it expands their utility for in vivo diagnostic, therapeutic or targeted drug delivery approaches [89]. In summary, these findings position the anti-GLAST-1 VHHs as valuable tools for mouse studies, while the cross-reactive anti-GLT-1 lead represents a promising candidate for future translational development.

Anti-GLT-1 binding to the human transporter was confirmed not only in overexpressing cell lines but also in hiPSC-derived astrocytes, a system in which GLT-1 expression more closely reflects endogenous levels and is commonly used to validate astrocytic identity during differentiation protocols [90,91]. However, detection of GLT-1 binding in this model required conversion of the monovalent VHH into a bivalent format. The use of different detection systems, i.e., a polyclonal anti-hIgG antibody for the VHH:Fc fusions while a monoclonal anti-FLAG antibody for the VHHs, might have played a role in improving signal amplification. In addition, lower endogenous GLT-1 levels most likely required avidity effects to allow target detection. Although hiPSC-derived astrocytes represent a more physiologically relevant model than overexpressing systems, they still present limitations: rarely do they fully resemble their adult human primary counterparts, often retaining a more fetal-like transcriptional profile [92]. In human astrocytes, GLT-1 expression has been described to be developmentally regulated and increased with maturation and co-culturing with neurons [93]. The astrocyte differentiation conditions used, i.e., presence of low serum that maintain cells in a slightly proliferative state together with the lack of multicellular signaling cues present in vivo, might have limited full astrocyte maturation [94,95] and, consequently, decreased GLT-1 expression. Beyond this, the intrinsic inter-replicate variability commonly associated with hiPSC-derived differentiation systems [96], might also explain the reduced GLT-1 protein levels and slightly different morphology observed for replicate #3, which led to a weaker VHH:Fc binding when compared with the other two replicates. Nevertheless, cross-reactivity represents an important finding, as it broadens VHH applications to human samples. The use of human tissues could support further validation of anti-GLT-1 VHH binding, while additional construct optimization, such as affinity maturation or multivalent engineering, may improve detection sensitivity in systems with low endogenous expression.

Anti-GLT-1 and GLAST-1 VHH target binding and astrocytic specificity were further evaluated ex vivo in mouse primary cells. Using mouse brain homogenates, we demonstrated that both anti-GLT-1 and anti-GLAST-1 VHHs preferentially bind to astrocytes, with over 60% of VHH-positive cells expressing astrocytic markers in all cases. This is consistent with the known enrichment of GLT-1 and GLAST-1 in astrocytes relative to other CNS cell types, such as microglia and oligodendrocytes [49]. The specificity of the assay was further supported by the expected oligodendrocyte-restricted binding of our previously validated anti-MOG VHH [37]. In addition, since GLAST-1 has been suggested to be susceptible to papain-mediated cleavage [97], we performed brain dissociations using both papain- and trypsin-based methods. Consistent with this, the anti-GLAST-1 VHH signal observed in trypsin-processed brain homogenates was completely absent in papain-treated samples, further supporting the specificity of all four VHHs for their intended target. Although neuronal binding cannot be fully excluded due to limitations of the dissociation protocol used, extensive transcriptomic [98,99] and proteomic [100,101] datasets consistently show that GLT-1 and GLAST-1 are predominantly expressed in astrocytes, supporting our interpretation.

We next evaluated whether these VHHs could detect their targets ex vivo in fixed brain tissues. Although it has been reported that VHHs might not be able to recognize certain proteins on fixed samples due to fixation-induced conformational changes or epitope masking [102,103], both anti-GLT-1 and anti-GLAST-1 VHHs produced robust neuropil staining that colocalized with commercial antibody signals and align with previously reported observations in early antibody characterization studies, identifying GLT-1 as the dominant glutamate transporter in forebrain astrocytic processes, and GLAST-1 as particularly highly enriched in Bergmann glia of the cerebellar molecular layer [63,64]. Some authors have reported evidence of a small fraction of GLT-1 being expressed in the synaptic terminals and neuronal axons in the rat hippocampus [104], and other studies identified three GLT-1 and GLAST-1 splice variants (a, b, c) with differential abundance and cellular distributions [105,106]. GLT1a and GLT1b have been reported to also be expressed in brain neurons [60], while GLT1c was found to be strongly expressed in neurons of the retina [107]. Accounting for these observations, complement studies using electron microscopy could help to determine whether our VHHs are pan or isoform specific, and if they exclusively bind to astrocytes or also to other cell types, i.e., neurons, within the brain or other tissues. Moreover, full binding characterization may benefit from the use of GLT-1 and GLAST-1 knock-out cell lines or mouse models to confirm target specificity and exclude off-target interactions.

Finally, to assess in vivo binding, VHHs were delivered directly into the mouse brain. For this purpose, peripheral administration was avoided due to the rapid renal clearance of native VHHs, resulting from their small size [108,109] and the limited ability of most VHHs to cross the BBB without engineering or targeting BBB receptors [110]. Intracerebroventricular or intracerebral delivery of VHHs have already been performed by several authors, demonstrating VHH brain presence several hours post-injection [34,38,111]. Twenty-four hours after intracerebral injection, both anti-GLT-1 and anti-GLAST-1 VHHs remained detectable, as revealed by immunofluorescence, confirming their stability and target engagement in the brain. Moreover, the absence of VHH signal in the contralateral hemisphere and in distant brain regions from the injection site argues against detection-antibody background. In contrast to anti-GLT-1 and GLAST-1 VHHs, the isotype control VHH (anti-β-lactamase) showed minimal retention, suggesting that target binding contributes to prolonged brain residence. This observation aligns with previous findings from our group showing that the incorporation of a brain-targeting VHH (i.e., anti-MOG) into multispecific antibodies increases their half-life in the CNS [37]. It is worth noting that, given the primarily qualitative nature of this experiment, only a single animal per group was analyzed at one time point. While this approach was sufficient for the exploratory objectives of the study, further experiments including larger cohort sizes and additional time points would help to validate and strengthen these observations.

The ability of anti-GLT-1 and anti-GLAST-1 VHHs to bind their targets in vivo highlights their potential as molecular tools for research, diagnostics, and potential astrocyte-targeted delivery in mouse models, with the cross-reactive GLT-1 VHH offering potential clinical relevance. As previously mentioned, the intrinsic properties of VHHs, such as their small size, rapid blood clearance, high tissue penetration, and ease of engineering, give them clear advantages over conventional antibodies for such applications. VHHs have already been successfully conjugated to radiotracers, fluorophores, and contrast agents for molecular imaging of brain tumors [112] and brain pathological protein aggregates [7]. Moreover, nanobody–drug conjugates are also being explored for targeted therapy, particularly in the oncology field [113,114,115]. Given the central role of astrocytes in numerous neurological disorders, VHHs targeting GLT-1 and GLAST-1 may serve as promising candidates for astrocyte-directed imaging or therapeutic strategies. In addition, although not demonstrated in this study, future experiments could benefit from the inherent modularity of VHHs, which would enable their fusion to BBB-shuttling VHHs, such as our anti-TfR VHH [21,23], as well as to other diverse therapeutic agents or delivery vehicles, opening the way to new promising non-invasive approaches for targeted systemic delivery to the brain.

## 5. Conclusions

Here, we describe and characterize the first VHHs against the astrocytic glutamate transporters GLT-1 or GLAST-1, all exhibiting apparent nanomolar affinities. Notably, the anti-GLT-1 VHH additionally cross-reacts with the human ortholog, an essential prerequisite for future translational or clinical development. Target engagement was confirmed across in vitro, ex vivo, and in vivo assays, and both sets of VHHs showed a clear tropism for astrocytes. Altogether, these findings position the developed VHHs not only as robust research tools for detecting GLT-1 and GLAST-1 but also for astrocyte identification. In addition, the anti-GLT-1 VHH might hold potential for cell-targeted delivery strategies within the CNS aimed at modulating astrocyte function in diverse neurological disease contexts.

## 6. Patents

The authors’ institution has filed a patent application protecting the VHH sequences disclosed in this manuscript and their potential applications. N.G.P., P.V.M., L.R. and M.D are listed as inventors. Sequences and corresponding reagents can be made available to academic researchers upon reasonable request and execution of an appropriate Material Transfer Agreement (MTA).

## Figures and Tables

**Figure 1:**
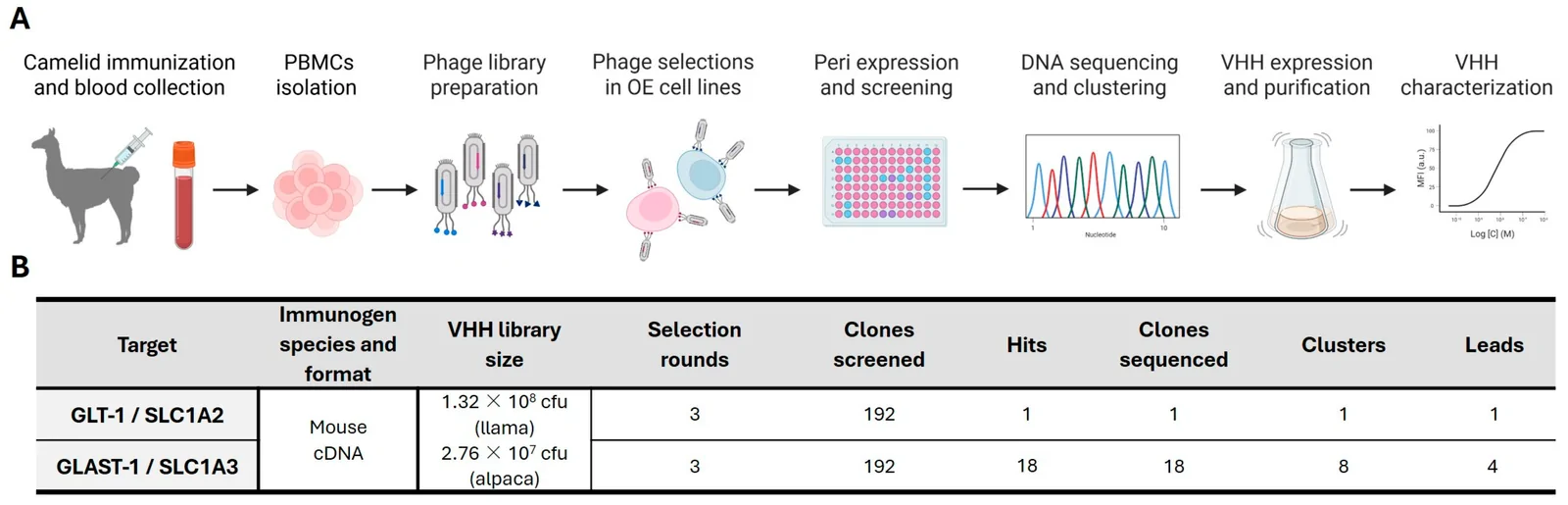
VHH generation and hit identification workflow. (**A**) Overview of VHH development protocol from camelid immunization to VHH expression and characterization. Created with Biorender.com. Peri: periplasmic extracts. (**B**) Summary of VHH library generation and screening process for lead identification.

**Figure 2:**
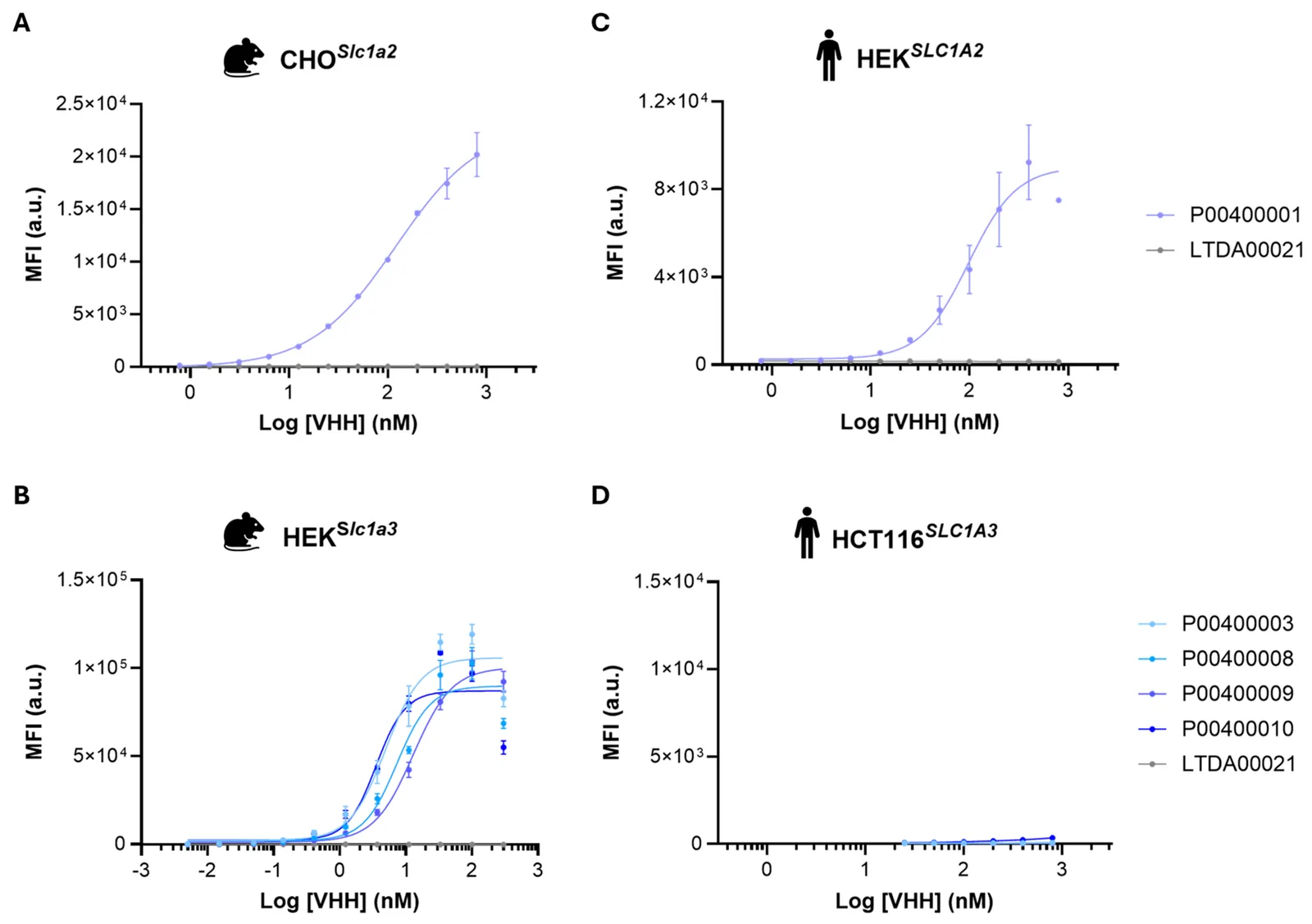
VHH binding curves to target overexpressing cell lines by flow cytometry. Anti-GLT-1 VHH (P00400001) binding to mouse (**A**) and human (**C**) GLT-1. Anti-GLAST-1 VHHs (P00400003/8/9/10) binding to mouse (**B**) and human (**D**) GLAST-1. (**A**–**D**) Anti-β-lactamase VHH (LTDA00021) was included as isotype control. Data represent mean ± SD (P00400001: *n* = 2; LTDA00021: *n* = 2–3; P0040003/8/9/10: *n* = 3; where ‘*n*’ represents technical replicates).

**Figure 3:**
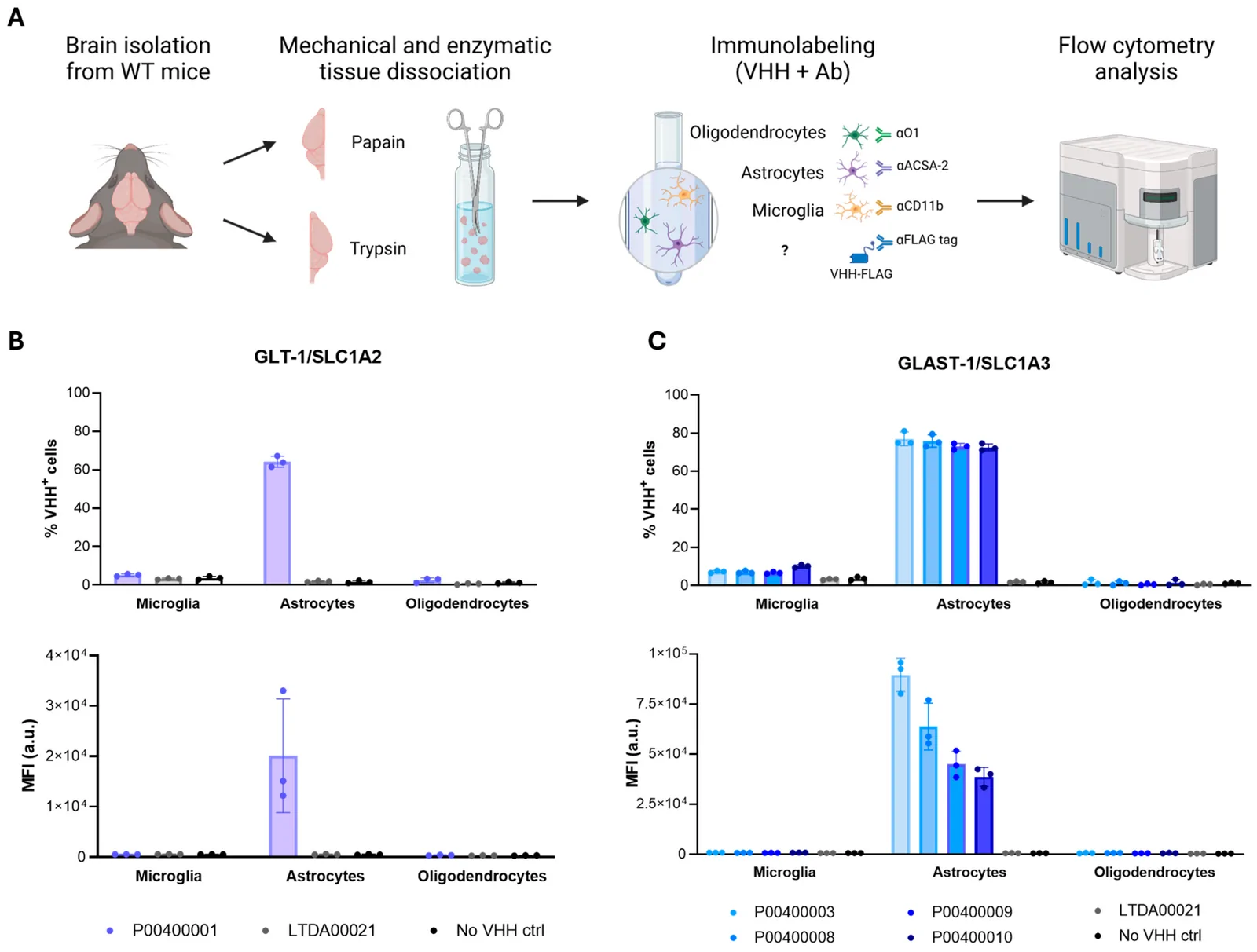
Anti-GLT-1 and GLAST-1 VHH ex vivo binding to mouse brain homogenates by flow cytometry. (**A**) Experimental design overview. Brain hemispheres of *n* = 3 WT C57BL/6 mice were dissociated mechanically and enzymatically (papain or trypsin) to obtain a single cell suspension followed by incubation with an antibody mix to detect microglia (CD11b), astrocytes (ACSA-2) and oligodendrocytes (O1), together with 100 nM of anti-GLT-1/GLAST-1/MOG/β-lactamase VHHs. Binding was assessed by flow cytometry. Created with Biorender.com. Anti-GLT-1 (**B**) and anti-GLAST-1 (**C**) VHHs binding to microglia, astrocytes and oligodendrocytes in trypsin-dissociated mouse brains. (**B**,**C**) Data represent % VHH^+^ cells ± SD from *n* = 3 (top graphs) or MFI of anti-FLAG-iF488 antibody ± SD from *n* = 3 (bottom graphs).

**Figure 4:**
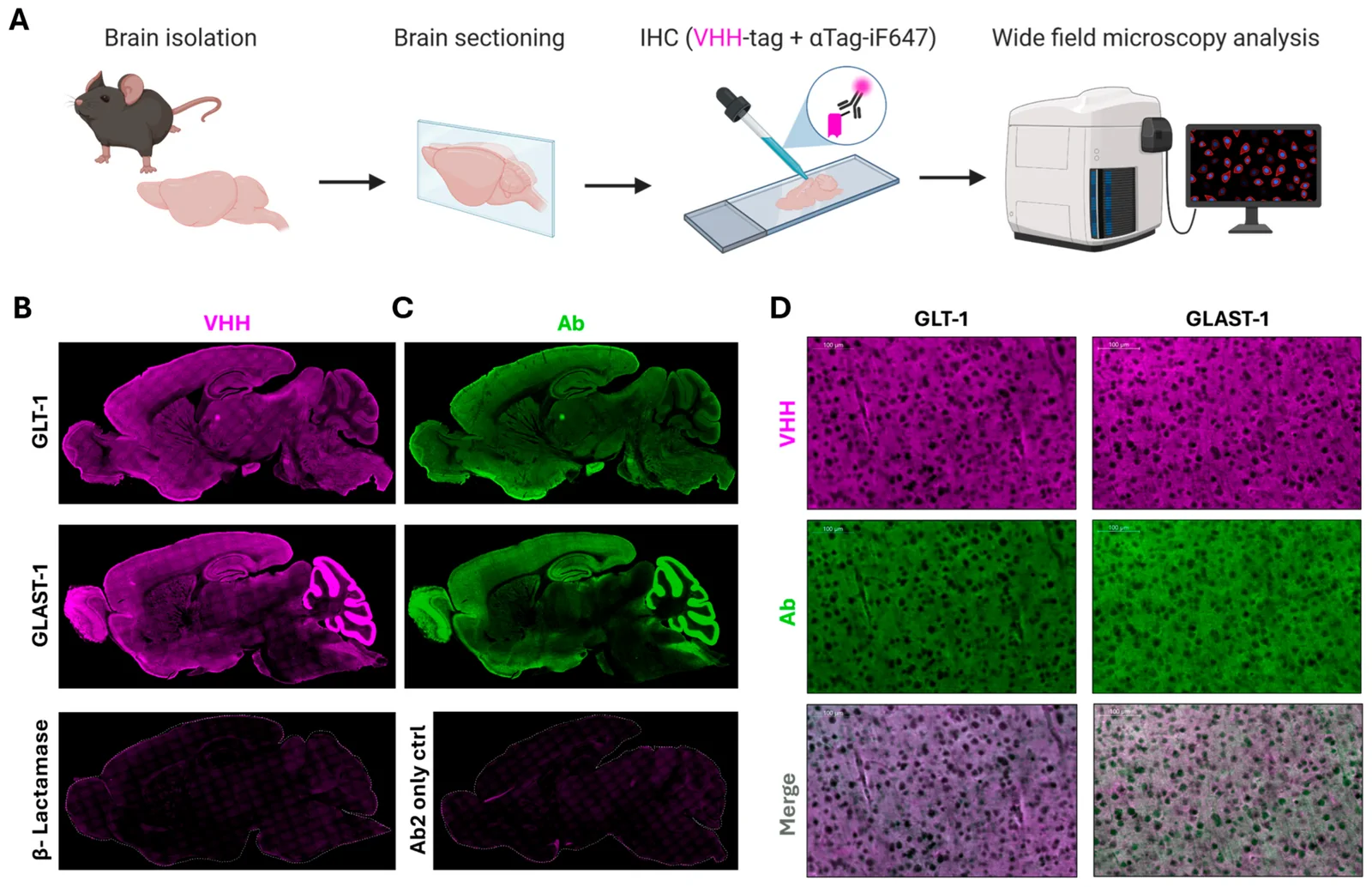
Anti-GLT-1 and GLAST-1 VHH ex vivo binding to mouse brain tissues by IHC. (**A**) Experimental design overview (created with Biorender.com). Brains from *n* = 3 WT C57BL/6 mice were sagittally sectioned and stained with 1 µM of anti-GLT-1/GLAST-1(P00400003)/β-lactamase VHHs (**B**) and anti-GLT-1/GLAST-1 commercial antibodies (Ab) (**C**). A mouse anti-HIS-iF647 antibody for VHH detection (Ab2). (**D**) Co-localization of anti-GLT-1/GLAST-1 VHHs (magenta) and commercial antibodies (green) in the cortex (100 µm). (**B**–**D**) Representative images are shown from *n* = 3/group, 2 x IHC sections/animal. Images were acquired with 20× objective using an Axioscan and visualized with ZEN 3.1 software (Zeiss).

**Figure 5:**
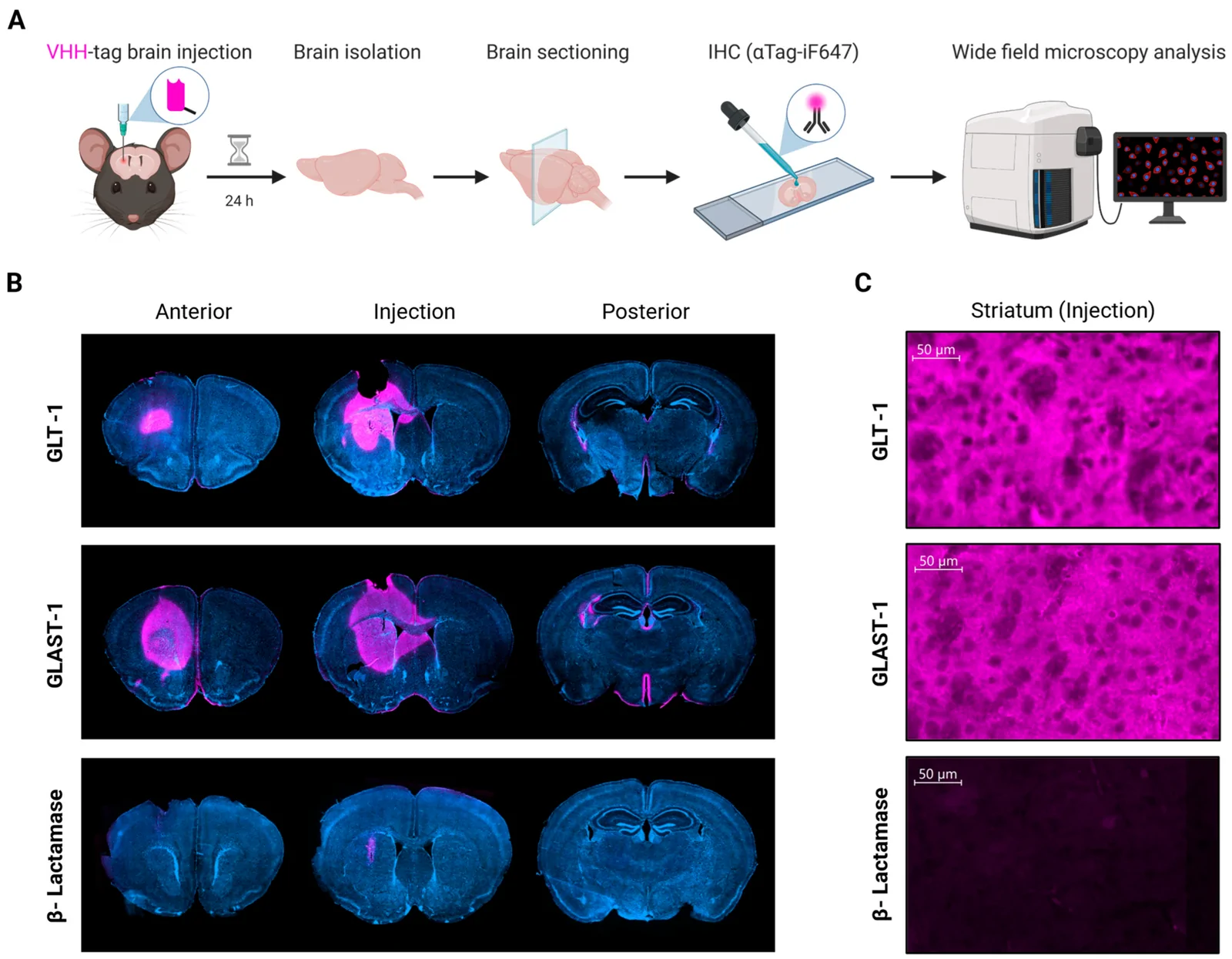
Anti-GLT-1 and GLAST-1 VHH in vivo binding in mouse brains. (**A**) Experimental design overview. VHHs were injected in *n* = 1 WT C57BL/6 mouse unilaterally in the striatum at 140 µM (~5 µg) and mice were sacrificed 24 h later. Brains were coronally sectioned and stained with a mouse anti-FLAG-iF647 antibody for VHH detection (magenta) and Hoechst for nuclear staining (blue). Created with Biorender.com. Anti-GLT-1, GLAST-1 and β-lactamase VHH (magenta) brain biodistribution (**B**) and staining pattern (scale bar at 50 µm) (**C**) 24 h post-injection. (**B**,**C**) Images were acquired with 20× objective using an Axioscan and visualized with ZEN 3.1 software.

**Table 1:** Overview of *Slc1a2* and *Slc1a3* selection criteria as VHH targets after database mining.

Gene	Protein	Brain Specificity (%) ^#^	Astrocyte Specificity in Brain (%) ^#^	Brain Expression (nTPM) ^#^	Homology Ms/Hu (%) *	Homology Ms/Alp (%) *
*Slc1a2*	GLT-1/EAAT2	84.6	81.9	6645.6	93.3	92
*Slc1a3*	GLAST-1/EAAT1	83.2	57.2	3647.7	94.2	94.9

Note: Represented data were obtained from the Human Protein Atlas (^#^) and Protein Database (PDB) processed with QIAGEN CLC Main Workbench version 23.0.4 (QIAGEN, Aarhus, Denmark) (*). Ms: mouse; Hu: human; Alp: alpaca. Homology was calculated considering only the extracellular domains of the transporters.

## Data Availability

The datasets generated in the current study are available from the corresponding authors upon reasonable request.

## Supplementary Materials

The following supporting information can be downloaded at https://www.mdpi.com/article/10.3390/antib15050089/s1. Figure S1: RNA-sequencing data for mouse and human *SLC1A2* and *SLC1A3* genes; Figure S2: GLT-1/EAAT2 and GLAST-1/EAAT1 topology and interspecies sequence homology [116]; Figure S3: Validation of mouse and human target overexpressing cells; Figure S4: Initial specificity and cross-reactivity assessment of anti-GLT-1 and GLAST-1 VHHs to mouse and human target overexpressing cell lines; Figure S5: Cross-reactivity and specificity assessment of anti-GLT-1 and GLAST-1 VHHs to mouse and human target overexpressing cell lines; Figure S6: GLT-1 expression and anti-GLT-1 VHH binding validation in hiPSC-derived astrocytes; Figure S7: Enrichment of astrocytes, oligodendrocytes and microglia from mouse brains and VHH binding to these cell populations using papain- and trypsin-based isolation methods; Figure S8: Enrichment of astrocytes, oligodendrocytes and microglia from mouse brains and VHH binding to these cell populations using papain and trypsin-based isolation methods. Table S1: Primary antibodies used for the detection of different brain cell populations by flow cytometry; Table S2: List of primary and secondary antibodies; Table S3: Anti-GLT-1 and anti-GLAST-1 VHHs affinities.

## Abbreviations

The following abbreviations are used in this manuscript:

AD	Alzheimer’s disease
ALS	Amyotrophic lateral sclerosis
AQP4	Aquaporin 4
BBB	Blood–brain barrier
CD98hc	CD98 heavy chain
cDNA	Complementary DNA
CHO	Chinese hamster ovary
CNS	Central nervous system
Dox	Doxycycline
EAAT1	Excitatory amino acid transporter 1
EAAT2	Excitatory amino acid transporter 2
FcR	Fragment crystallizable receptor
GFAP	Glial fibrillary acidic protein
GLAST-1	Glutamate aspartate transporter 1
GLT-1	Glutamate transporter 1
GOI	Gene of interest
GS	Glutamine synthetase
HCT116	Human colorectal carcinoma cell line
HD	Huntington’s disease
HEK	Human embryonic kidney
HPA	Human protein atlas
IHC	Immunohistochemistry
IGF1R	Insulin-like growth factor 1 receptor
iPSC	Induced pluripotent stem cell
KO	Knock-out
MFI	Median fluorescence intensity
MOG	Myelin oligodendrocyte glycoprotein
MS	Multiple sclerosis
OE	Overexpression
PBMC	Peripheral blood mononuclear cell
PCR	Polymerase chain reaction
PD	Parkinson’s disease
RMT	Receptor-mediated transcytosis
scRNA	Single-cell RNA
TfR	Transferrin receptor
VHH	Variable domain of heavy-chain antibody
VHH:Fc	VHH fused to Fc region
WB	Western blot
WT	Wild type
