# Supplementary material for: Development and Validation of Novel VHHs Targeting Astrocyte-Enriched Glutamate Transporters GLT-1 and GLAST-1
Source: Antibodies (Basel). 2026 Sep 29;15(5):89. doi: 10.3390/antib15050089 (PMC13700591; doi:10.3390/antib15050089)
Supplement: Supplementary file 1 [file antibodies-15-00089-s001.zip › antibodies-4429563-supplementary.pdf]

Supplementary Materials

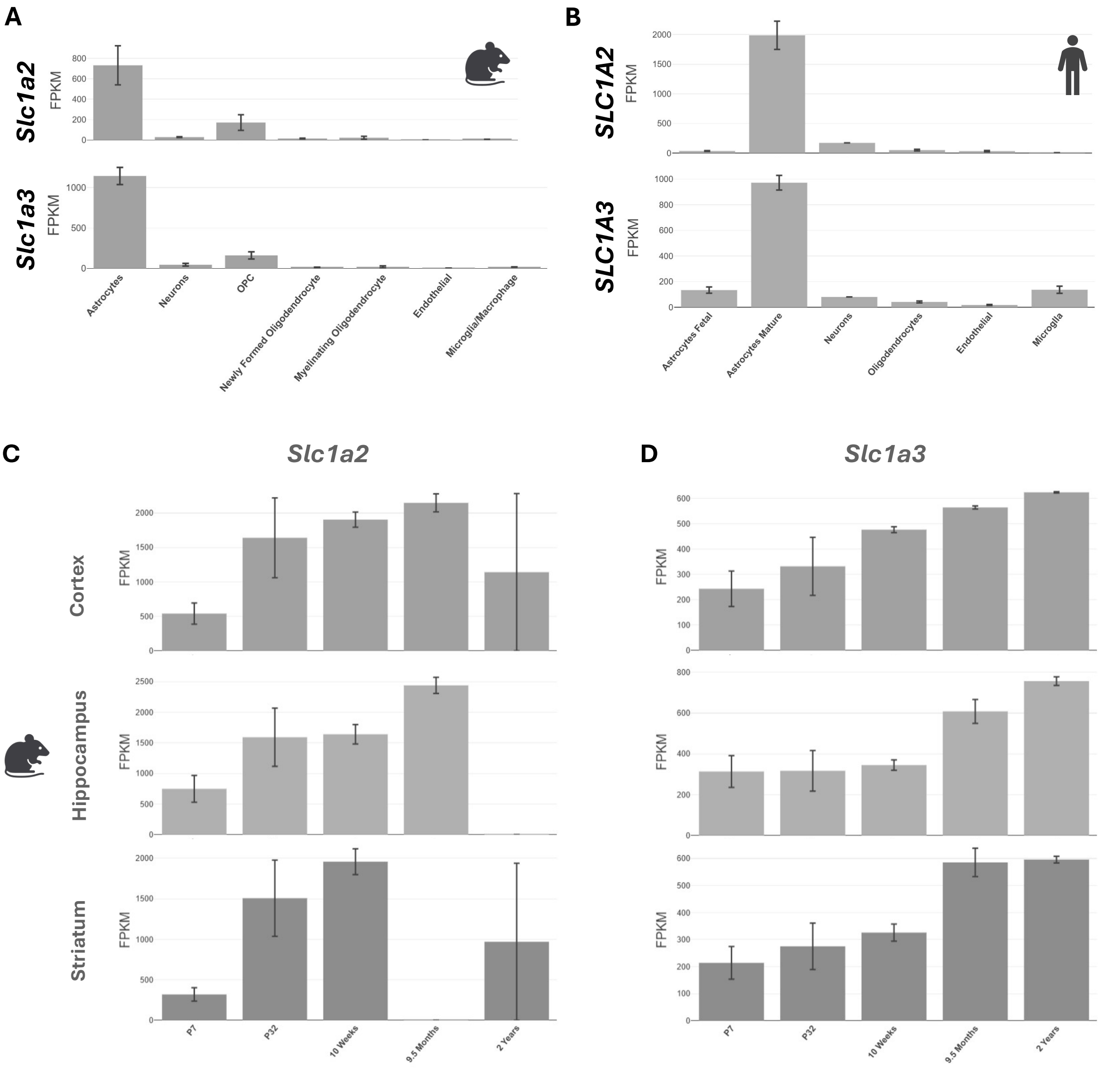

Supplementary Figure S1. **RNA-sequencing data for mouse and human *SLC1A2* and *SLC1A3* genes.** Expression levels of *Slc1a2* and *Slc1a3* in mouse **(A)** and human **(B)** across different brain cell populations. This data contributed to target selection for VHH development to detect astrocytes. *Slc1a2* **(C)** and *Slc1a3* **(D)** expression in mouse cortex, hippocampus and striatum at different ages**. (A, B)** This data was used to decide mouse age for the different *in vivo* experiments (~10 weeks) and **(C, D)** brain region for VHH local injection (striatum). **(A–D)** RNA-sequencing data is indicated as Fragments Per Kilobase Million (FPKM). Figure was extracted and adapted from: Brain RNA-Seq – Brain RNA-Seq).

SLC1A2 / EAAT2 / GLT-1	SLC1A3 / EAAT1 / GLAST-1

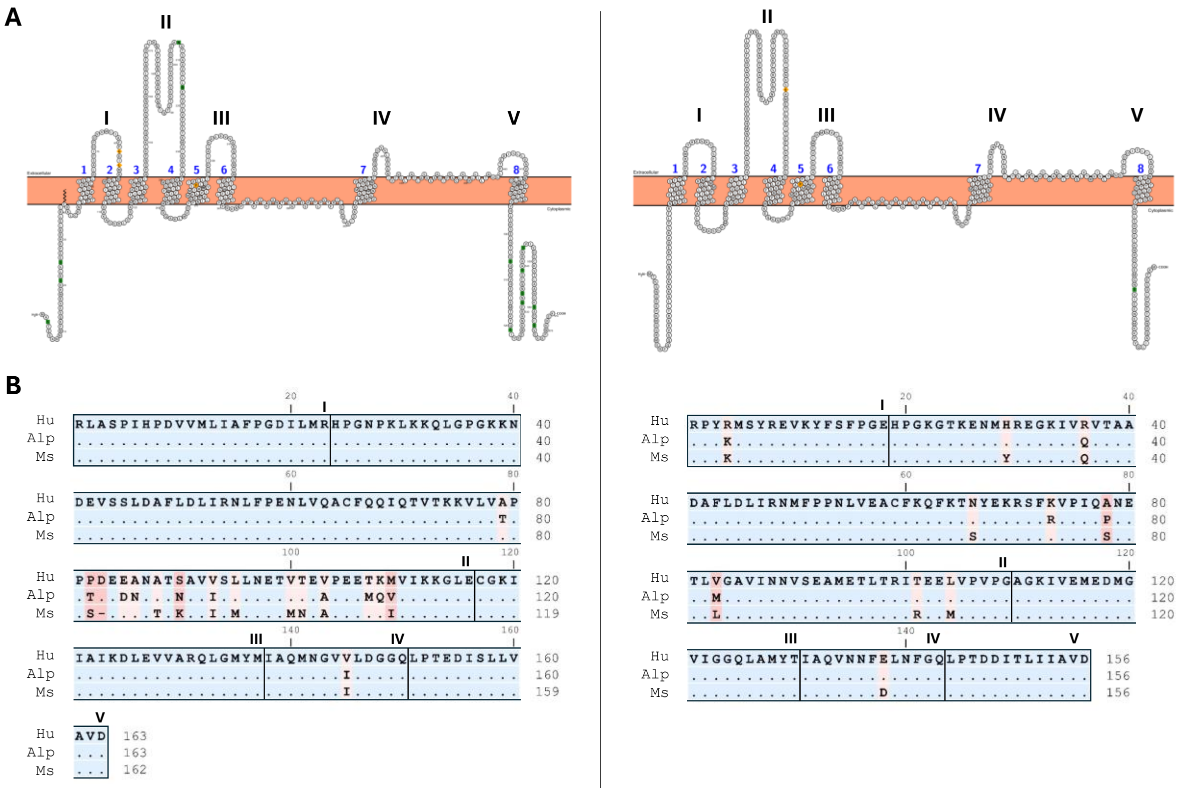

C

	SLC1A2 / EAAT2 / GLT-1					
	Overall seq identity			EC loops seq identity		
	Hu	*Alp*	*Ms*	*Hu*	*Alp*	*Ms*
Hu		96.5%	95.1%		93.3%	93.3%
Alp	20		95.3%	11		92%
Ms	28	27		11	13	

	SLC1A3 / EAAT1 / GLAST-1					
	Overall seq identity			EC loops seq identity		
	Hu	*Alp*	*Ms*	*Hu*	*Alp*	*Ms*
Hu		97.8%	96.5%		96.8%	94.2%
Alp	12		96.3%	5		94.9%
Ms	19	20		9	8	

D

	GLT-1 vs. GLAST-1	
	Overall seq identity	EC loops seq identity
Hu	47.4%	39.8%
Ms	47.6%	39.6%

Supplementary Figure S2. GLT-1/EAAT2 and GLAST-1/EAAT1 topology and interspecies sequence homology. (A) Schematic representation of human GLT-1/EAAT2 and GLAST-1/EAAT1 topology in the cell membrane. Latin numerals represent transmembrane domains (*1-8*) and roman numerals, extracellular (EC) loops (*I-V*). Figures were generated with Protter - interactive protein feature visualization (https://doi.org/10.1093/bioinformatics/btt607). **(B)** EC domains sequence alignment between human (Hu), alpaca (Alp) and mouse (Ms) orthologs for both GLT-1 (*left*) and GLAST-1 (*right*). Different EC loops are indicated in boxes with roman numerals (*I-V*). Matching residues are indicated with dots and highlighted *in blue*, while differences in the aminoacidic sequence are highlighted *in red*. **(C)** Overall and EC domains sequence identity between Hu, Alp and Ms orthologs for both GLT-1 (*left*) and GLAST-1 (*right*). Numbers represent percent sequence identify (*in blue*) and sequence differences (*in red*). (**D**) Overall and EC domains sequence homology between Hu and Ms GLT-1 and GLAST-1. (**B, C, D**) Protein alignments and interspecies pairwise comparisons were performed using CLC software (Qiagen). (**A-D**) All protein sequences were extracted from Uniprot: GLT-1 (human: P43004; mouse: P43006; alpaca: A0A6J3AUR2); GLAST-1 (human: P43003; mouse: P56564; alpaca: A0A6I9IDN1).

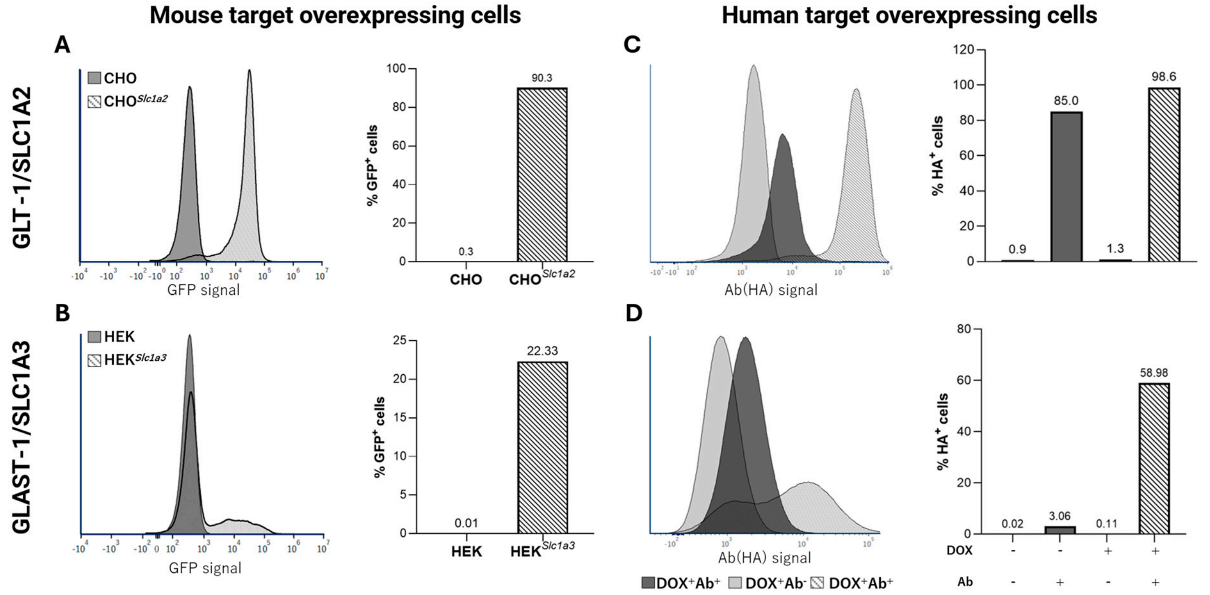

Supplementary Figure S3. Validation of mouse and human target overexpressing cells. Quality control of overexpression of mouse *Slc1a2* on CHO Flp-In™ cells **(A)** or *Slc1a3* on HEK cells **(B)** using GFP fluorescent signal by flow cytometry. Quality control of overexpression of human *SLC1A2* on Dox- or non-induced HEK^{SLC1A2} WT-OE cells **(C)** or *SLC1A3* on Dox- or non-induced HCT116^{SLC1A3} KO-OE cells **(D)** using HA tag detection by flow cytometry. A mouse anti–HA tag was used as primary antibody (1:200, Ca. No. 901515, Biolegend) and detected using a goat anti–mouse IgG-AF647 (1:500, Cat. No. A-21236, Invitrogen). **(A–D)** Histograms show area of fluorescent (GFP or HA) intensity *(left graphs*) and bar graphs % of GFP⁺ or HA⁺ cells *(right graphs).*

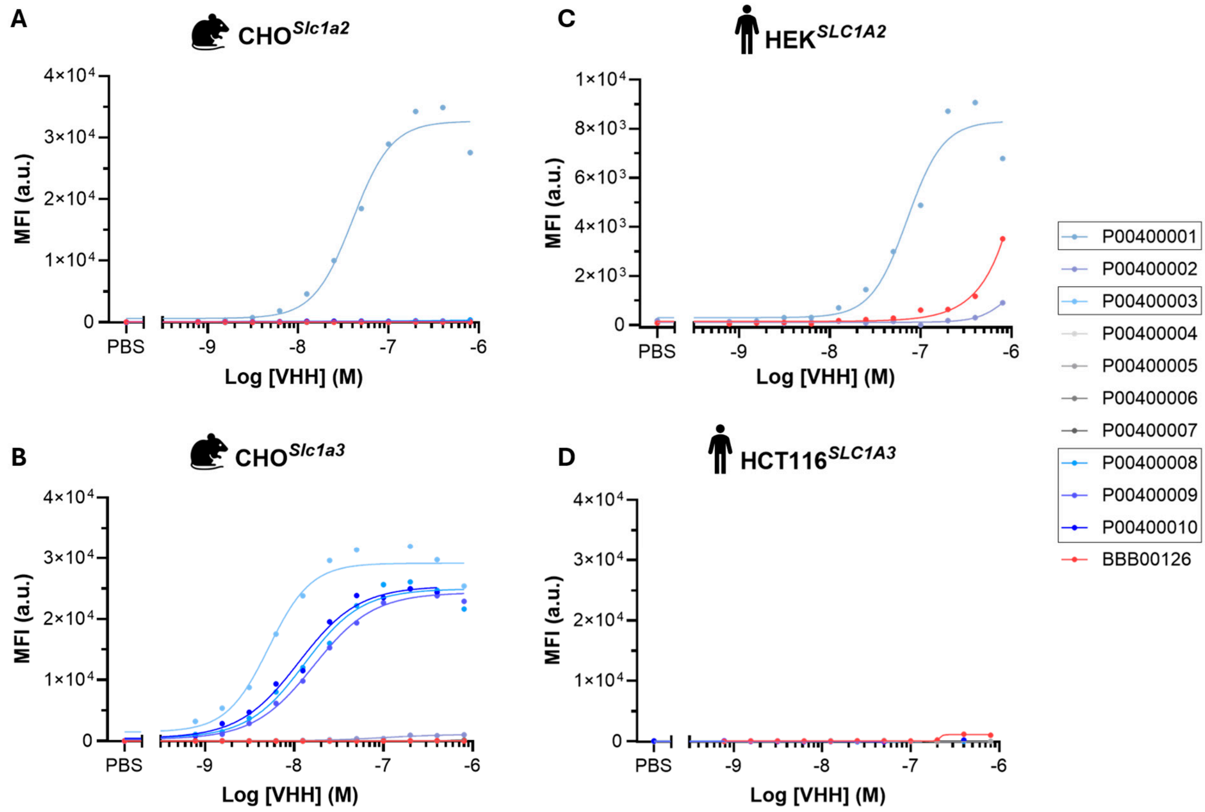

Supplementary Figure S4. Initial specificity and cross-reactivity assessment of anti–GLT-1 and GLAST-1 VHHs to mouse and human target overexpressing cell lines. Anti–GLT-1 and –GLAST-1 VHH binding to mouse **(A)** and human **(C)** GLT-1 (SLC1A2) and mouse **(B)** and human **(D)** GLAST-1 (SLC1A3) overexpressing cell lines. **(A–D)**

Anti-GFP VHH (BBB00126) was included as isotype control. Data represents MFI from n = 1, where '*n*' represents technical replicates. Binding VHHs are indicated with a square in the graphs' legend.

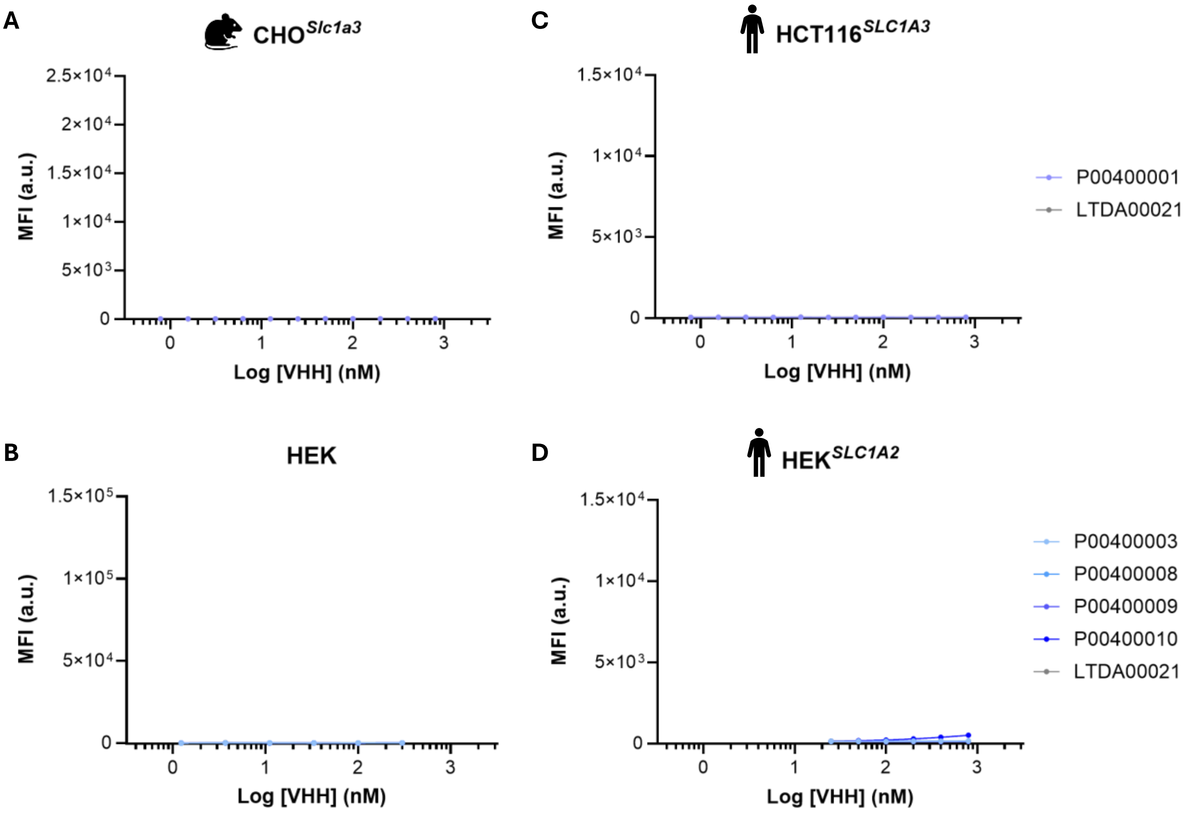

Supplementary Figure S5. Cross-reactivity and specificity assessment of selected anti–GLT-1 and –GLAST-1 VHHs to mouse and human target overexpressing cell lines. Anti–GLT-1 VHH binding to mouse **(A)** and human **(C)** GLAST-1 overexpressing cell lines. Anti–GLAST-1 VHH binding to non-overexpressing **(B)** and human GLT-1 overexpressing **(D)** cells. **(A–D)** Anti–β-lactamase VHH (LTDA00021) was included as isotype control. Data represents mean ± SD from n = 3, where 'n' represents technical replicates. **Note:** the flat binding curves observed in panels A-C reflect background-level fluorescence and demonstrate the absence of detectable cross-reactivity under the tested conditions, maintaining the same MFI scale as in Figure 2.

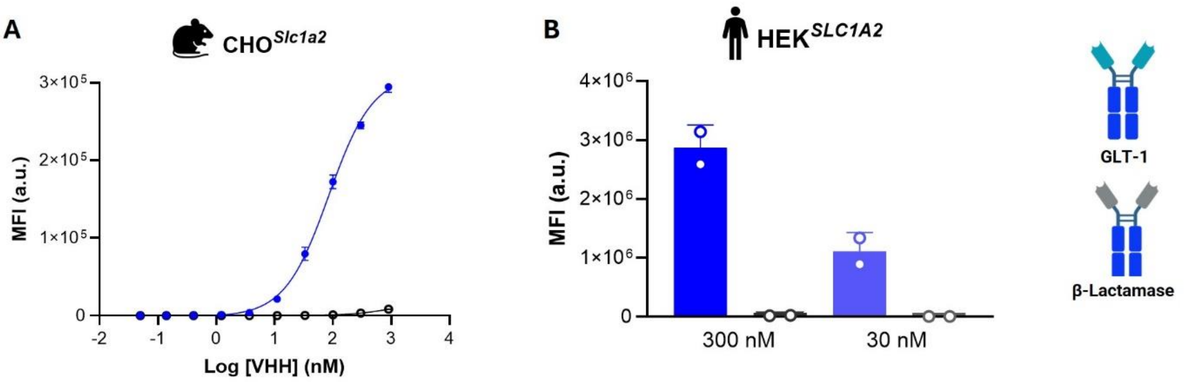

Supplementary Figure S6. Validation of VHH:Fc binding to GLT-1. VHH:Fc binding to GLT-1 was confirmed in CHO^{Slc1a2} cells for the mouse **(A)** and in HEK^{SLC1A2} cells for the human **(B)** transporter. **(A-B)** Anti–β-lactamase VHH:Fc was included as isotype control. Anti-human IgG-AF647 was used for VHH:Fc detection. Data represents mean ± SD from n = 2-3, where 'n' represents technical replicates.

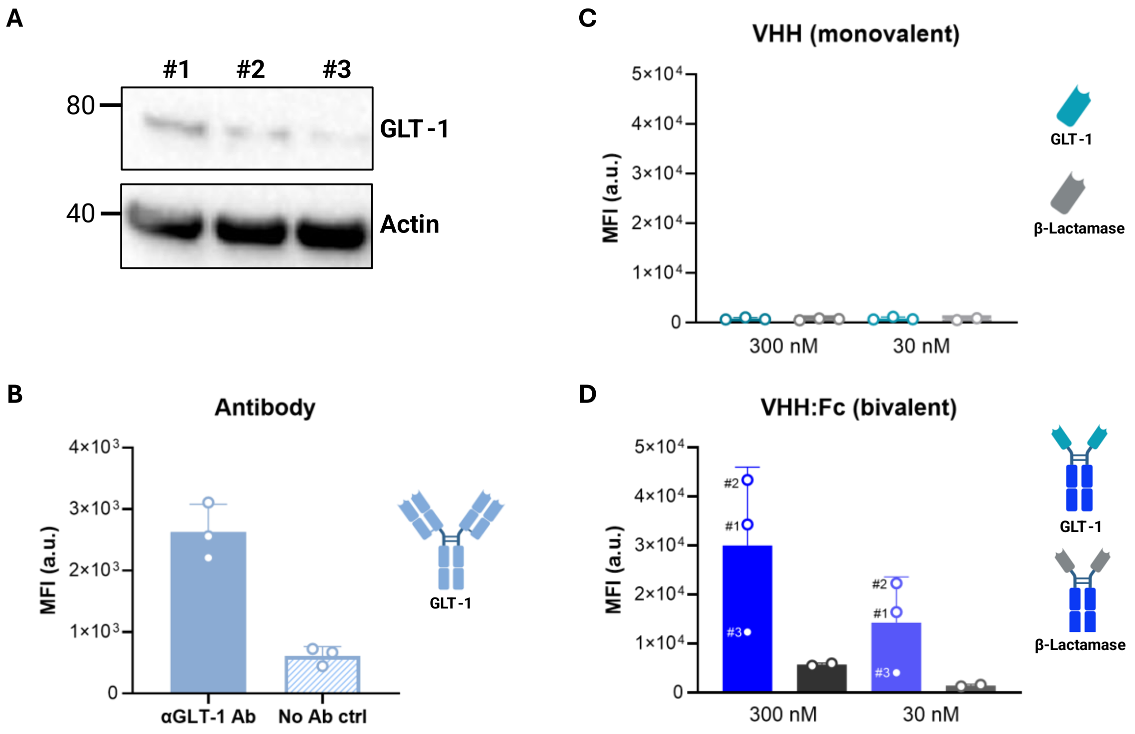

Supplementary Figure S7. GLT-1 expression and anti–GLT-1 VHH binding validation in hiPSC-derived astrocytes. Validation of GLT-1 protein expression on hIPSC by WB **(A)** or flow cytometry **(B)**. Anti–GLT-1 VHH **(C)** or VHH:Fc **(D)** binding on hiPSC astrocytes by flow cytometry. **(B, C, D)** Data represents mean ± SD from n = 2-3, where *'n'* corresponds to independent differentiations.

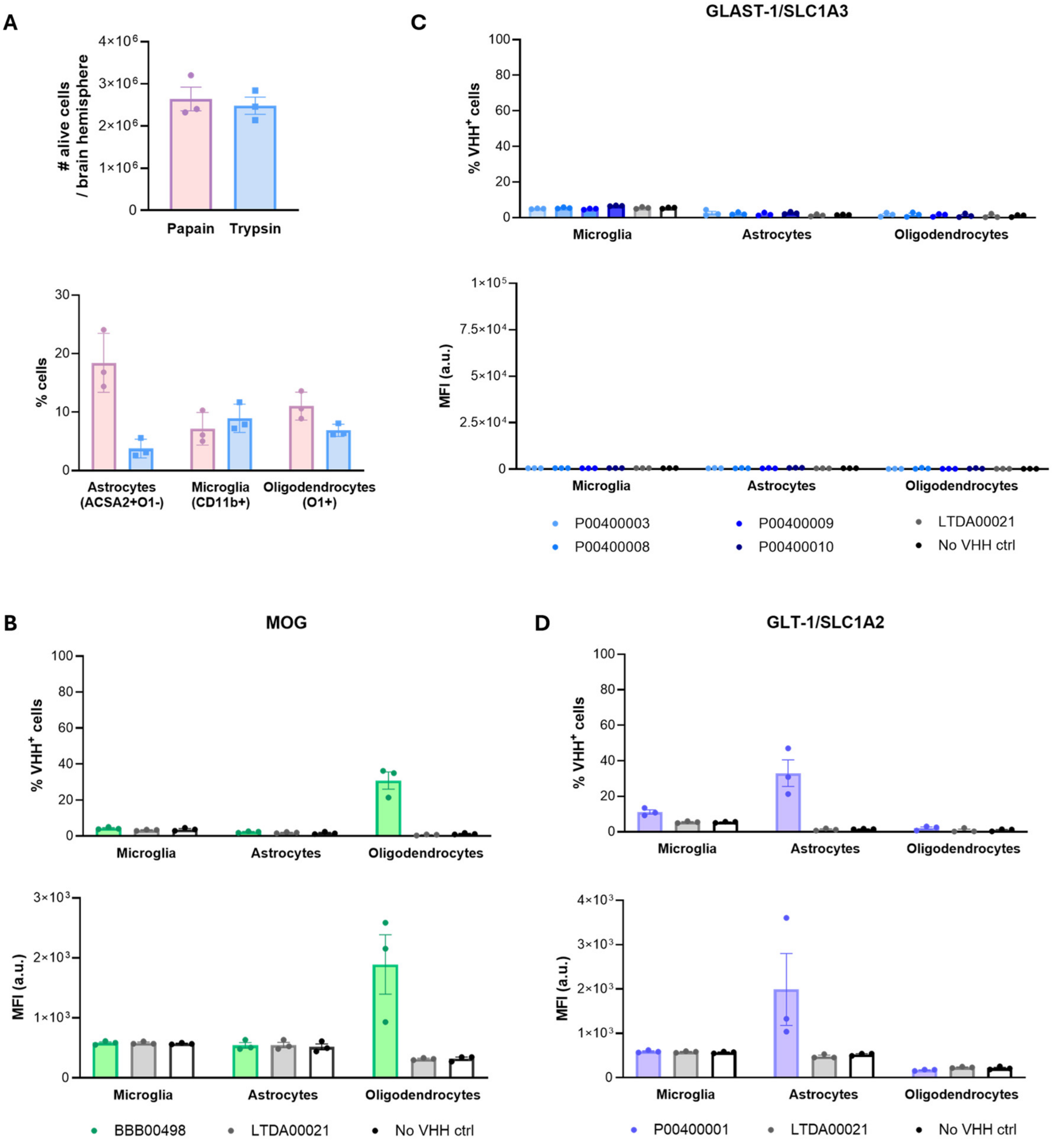

Supplementary Figure S8. Enrichment of astrocytes, oligodendrocytes and microglia from mouse brains and VHH binding to these cell populations using papain and trypsin-based isolation methods. (A) # viable cells isolated from hemibrains *(top graph)* and % of astrocytes, microglia or oligodendrocytes *(bottom graph)* using papain or trypsin-based methods. **(B)** anti–MOG VHH binding in trypsin-dissociated samples. Anti–GLAST-1 **(C)** or anti–GLT-1 **(D)** VHH binding in papain-dissociated samples. **(B, C, D)** % VHH^{+} cells *(top graphs)* or anti–FLAG-iF488 MFI *(bottom graphs)*.

Supplementary Table S1. Primary antibodies used for the detection of different brain cell populations in mouse brain homogenates by flow cytometry.

Cell type	*Target*	*Antibody*
Astrocytes	***ACSA-2***	**ACSA-2-PE**<br>(1:50, Cat. No.130-116-244, Miltenyi Biotech)
Microglia	***CD11b***	**CD11b-BV421**<br>(1:500, Cat. No. 562605, BD Biosciences, Franklin Lakes, New Jersey, USA)
Oligodendrocytes	***O1***	**O1-eF660**<br>(1:800, Cat. No. 50-6506-82, Thermo Fisher)

Supplementary Table S2. **List of primary and secondary antibodies.**

Target	*Primary Antibody*	*Secondary Antibody*
VHH	**Mouse anti–HIS-iF647**[1]<br>(1:500, Cat. No. A01802-100, Genscript)<br>**Mouse anti–FLAG-iF647**[1,2]<br>(1:500, Cat. No. A01811-100, Genscript)<br>**Mouse anti–FLAG-iF488**[2]<br>(1:500, Cat. No. A01809, Genscript)	None
VHH:Fc	None	**Goat anti–human IgG(H+L)-AF647**[2]<br>(1:500, Cat. No. A21236, Thermo Fisher)
GLT-1	**Rabbit anti–EAAT2/GLT1**[1]<br>(1:200, Cat. No. NBP1-20136, Novus Biologics)	**Goat anti–rabbit IgG (H+L)-AF488**[1]<br>(1:500, Cat. No. A-11008, Thermo Fisher)<br>**Goat anti–rabbit IgG (H+L)-AF647**[2]<br>(1:500, Cat. No. A21244, Thermo Fisher)
	**Rabbit anti–EAAT2/GLT1**[3]<br>(1:1000, Cat. No. GLT11-S, Alpha Diagnostic)	**Goat anti–rabbit-HRP**[3]<br>(1:3000, Cat. No. 1706515, Biorad)
GLAST-1	**GLAST (ACSA-1)-PE**[1]<br>(1:200, Cat. No. 130-118-483, Miltenyi Biotech)	None

Note: used for [1]immunohistochemistry; [2]flow cytometry; [3]Western Blot.

Supplementary Table S3. Anti-GLT-1 and anti-GLAST-1 VHHs affinities.

Target	*VHH*	*Mouse EC_{50} (nM)*	*Human EC_{50} (nM)*
GLT-1 / SLC1A2	P00400001	121.4	98.3
GLAST-1 / SLC1A3	P00400003	4.9	No binding
	P00400008	7.3	No binding
	P00400009	12.9	No binding
	P00400010	3.6	No binding

Note: EC_{50} values were obtained from binding curves on overexpressing cells by flow cytometry.
